# Strategic design of Fe and N co-doped hierarchically porous carbon as superior ORR catalyst: from the perspective of nanoarchitectonics†

**DOI:** 10.1039/d2sc02726g

**Published:** 2022-09-01

**Authors:** Minjun Kim, Konstantin L. Firestein, Joseph F. S. Fernando, Xingtao Xu, Hyunsoo Lim, Dmitri V. Golberg, Jongbeom Na, Jihyun Kim, Hiroki Nara, Jing Tang, Yusuke Yamauchi

**Affiliations:** Australian Institute for Bioengineering and Nanotechnology (AIBN), School of Chemical Engineering, The University of Queensland Brisbane Queensland 4072 Australia y.yamauchi@uq.edu.au; Centre for Materials Science, School of Chemistry and Physics, Queensland University of Technology (QUT) 2 George Street Brisbane Queensland 4000 Australia; International Center for Materials Nanoarchitectonics (WPI-MANA), National Institute for Materials Science (NIMS) 1-1 Namiki Tsukuba Ibaraki 305-0044 Japan nara.hiroki@nims.go.jp; New & Renewable Energy Research Center, Korea Electronics Technology Institute (KETI) 25, Saenari-ro, Bundang-gu Seongnam-si Gyeonggi-do 13509 Republic of Korea; Materials Architecturing Research Center, Korea Institute of Science and Technology 5 Hwarang-ro 14-gil, Seongbuk-gu Seoul 02792 Republic of Korea; Solar Energy R&D Department, Green Energy Institute Mokpo Jeollanamdo 58656 Republic of Korea; School of Chemistry and Molecular Engineering, Shanghai Key Laboratory of Green Chemistry and Chemical Processes, East China Normal University Shanghai 200062 China jingtang@chem.ecnu.edu.cn

## Abstract

In this study, we present microporous carbon (MPC), hollow microporous carbon (HMC) and hierarchically porous carbon (HPC) to demonstrate the importance of strategical designing of nanoarchitectures in achieving advanced catalyst (or electrode) materials, especially in the context of oxygen reduction reaction (ORR). Based on the electrochemical impedance spectroscopy and ORR studies, we identify a marked structural effect depending on the porosity. Specifically, mesopores are found to have the most profound influence by significantly improving electrochemical wettability and accessibility. We also identify that macropore contributes to the rate capability of the porous carbons. The results of the rotating ring disk electrode (RRDE) method also demonstrate the advantages of strategically designed double-shelled nanoarchitecture of HPC to increase the overall electron transfer number (*n*) closer to four by offering a higher chance of the double two-electron pathways. Next, selective doping of highly active Fe–N_*x*_ sites on HPC is obtained by increasing the nitrogen content in HPC. As a result, the optimized Fe and N co-doped HPC demonstrate high ORR catalytic activity comparable to the commercial 20 wt% Pt/C in alkaline electrolyte. Our findings, therefore, strongly advocate the importance of a strategic design of advanced catalyst (or electrode) materials, especially in light of both structural and doping effects, from the perspective of nanoarchitectonics.

## Abstract 2

This study elucidates the role of each class of nanopore by in-depth electrochemical analysis of three types of ZIF-8-derived carbons. Also, engineered co-doping of Fe and N is found essential to selectively form Fe–N_*x*_ sites in the carbon matrix.

## Graphical abstract

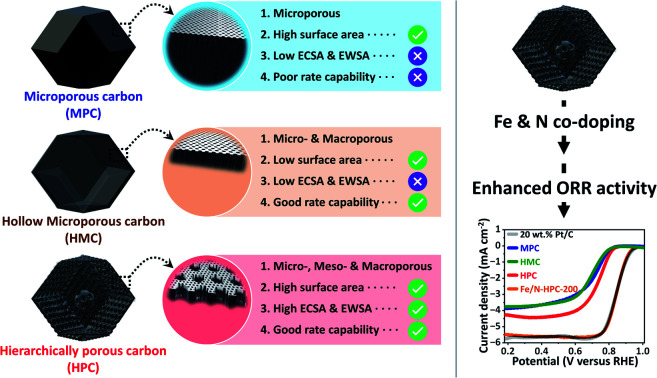

## Introduction

The efficiency of current state-of-the-art energy-related applications is heavily influenced by the type of catalyst (or electrode) materials that can effectively reduce the activation energy or modulate the reaction mechanisms to promote the desired chemical reactions. Particularly, sluggish oxygen reduction reaction (ORR) still presents a major challenge in fuel cells (FCs) and metal–air batteries (MABs), hence limiting the scope of choice for catalysts to Pt-group metal (PGM) species.^1–3^ Nevertheless, high production cost, low earth-abundance and low stability of PGM species advocate the need for novel ORR catalysts with cheap and earth-abundant metals.^2–5^ As a result, there have been significant research efforts to adopt novel synthetic strategies to obtain PGM-free ORR catalysts.

Various traditional porous materials (*e.g.*, mesoporous silica, zeolites, metal–organic frameworks (MOFs), *etc.*) are often not suitable as catalyst (or electrode) materials for electrochemical applications due to the lack of electrical conductivity and chemical stability. To confer electrical conductivity and stability, a direct-carbonization process is commonly used for certain porous materials to convert them into porous carbon materials. For instance, a type of MOF, zeolitic imidazolate framework-8 (ZIF-8), can be thermally converted into microporous carbons with high specific surface areas.^6,7^ Along with carbon materials such as carbon nanotube (CNT), graphene, and porous carbons,^8–11^ MOF-derived carbons are actively investigated as alternative materials to replace expensive PGM species.^12,13^ Ideally, carbon materials should possess intrinsic chemical compositions, such as nitrogen (N), sulfur (S), boron (B), and phosphorus (P), that can directly contribute to ORR catalytic activity or coordinate with transition metals to form more active catalytic sites.^14–18^ As ZIF-8 possesses rich N content and high surface area, it has been heavily exploited to synthesize PGM-free ORR catalysts.^19^ Despite high specific surface area, moderate electrical conductivity, and N-rich nature, however, the electrochemical performance of ZIF-8 derived carbons is still largely limited by its highly microporous structure restricting efficient diffusion of substances.^20,21^ This, in turn, leaves a significant portion of its surface area electrochemically redundant. To avoid such undesirable loss of surface area in electrochemical reactions, the design of nanoarchitecture of catalyst (or electrode) materials must be considered in a more strategic way to expose as much surface area as possible to the surrounding electrochemical environment.^22–26^

Herein, we first prepare hollow microporous carbon (HMC, involving micro- and macropores) and hierarchically porous carbon (HPC, involving micro-, meso- and macropores) by direct-carbonization of the modified ZIF-8.^20,27–29^ Electrochemical behaviors of both samples are then compared to that of ZIF-8 derived microporous carbon (MPC, mainly involving micropores) to carefully examine the effect of different nanoarchitectures in the context of ORR. Based on electrochemical impedance spectroscopy (EIS) and ORR studies, the following things are identified: (1) micropores maximize specific surface area but severely restrict both electrochemical wettability and accessibility. (2) Mesopores significantly relieve the diffusion restriction, hence improving electrochemical wettability and accessibility. (3) Macropore significantly reduces specific surface area but typically contributes to the rate capability of the porous carbon materials. In addition, the results of the rotating ring disk electrode (RRDE) method also demonstrate the advantages of strategically designed double-shelled nanoarchitecture of HPC to increase the overall electron transfer number (*n*) closer to four by offering a higher chance of the double two-electron pathways.

Next, we extend the scope of this study to the control of the local atomic environment of the porous carbon to enhance ORR catalytic activity.^30–33^ Among PGM-free catalysts, M–N_*x*_ (where M includes transition metal species) catalytic sites show promising ORR catalytic activity. M–N_*x*_ sites are known to offer energetically favorable adsorption sites for ORR intermediates (O*, OH* and OOH*), therefore, serving as excellent ORR catalytic sites.^34–38^ Fe–N–C catalysts, for example, possess ORR catalytic activity comparable to that of Pt/C, while having greater electrochemical stability than Pt/C in both alkaline and acidic electrolytes. Prior to the synthesis of a high-performance Fe–N–C catalyst, the following physical and chemical aspects must be carefully considered: (1) high density of Fe–N_*x*_ moieties in the carbon framework should be achieved to increase ORR kinetics. (2) Catalytic sites should be well-dispersed and easily accessible/escapable by reactants/products. (3) Catalyst should possess good electrical conductivity to transfer electrons efficiently towards the catalytic sites.^31–33^ To meet the criteria, we conduct Fe doping on our best-performing N-doped carbon scaffold, namely HPC, to form Fe–N_*x*_ sites.^39–43^ It is clearly found that enrichment of HPC with more N atoms leads to the enhancement of ORR catalytic activity by forming more Fe–N_*x*_ sites during the thermal treatment. Indeed, the optimized Fe, N co-doped HPC successfully achieves highly comparable ORR catalytic activity (*E*_onset_ = 0.96, *E*_1/2_ = 0.85 and *n* = 3.97) to the commercial 20 wt% Pt/C (*E*_onset_ = 0.97, *E*_1/2_ = 0.85 and *n* = 3.97) and superior stability performance in alkaline electrolyte.

## Results and discussion

**Fig. 1:**
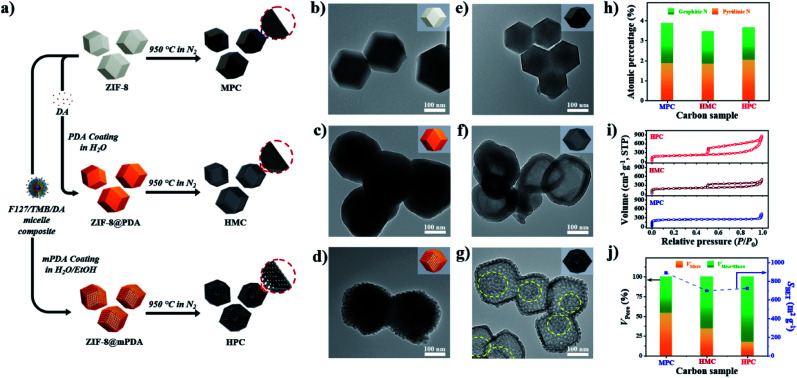
(a) Description of synthesis pathways from ZIF-8 to ZIF-8@PDA and ZIF-8@mPDA, and direct-carbonization to MPC, HMC and HPC, respectively. TEM images of (b) ZIF-8, (c) ZIF-8@PDA, (d) ZIF-8@mPDA, (e) MPC, (f) HMC, and (g) HPC. (h) Atomic percentage of graphitic and pyridinic nitrogen species in MPC, HMC and HPC. (i) Nitrogen adsorption/desorption isotherms and (j) the ratio of pore volume between micropore (<2 nm) and meso-/macropore (>2 nm) (left *y*-axis) and *S*_BET_ (right *y*-axis) of MPC, HMC and HPC.

As described in Fig. 1a, nonporous polydopamine (PDA) and mesostructured polydopamine (mPDA) coatings were applied on the surface of ZIF-8 particles. The detailed procedures are given in the Experimental Section in ESI.† Typically, ZIF-8 particles of ∼200 nm in diameter were coated to obtain ZIF-8@PDA or ZIF-8@mPDA, respectively (Fig. 1b–d and S1a–c†). Unlike ZIF-8@PDA, which was obtained by simple PDA coating on ZIF-8, ZIF-8@mPDA was synthesized by implementing F127 and 1,3,5-trimethylbenzene (TMB) as the soft-template (Fig. 1a). For the successful coating of mPDA, the role of TMB is critical as it is essential to form the soft-template. When the PDA coating was conducted without TMB, ZIF-8 particles coated with numerous PDA nanospheres were obtained (Fig. S2†). This is because F127 molecules are not able to form micelles without the help of TMB to stabilize the hydrophobic core. Changes in the surface elemental compositions after the coating are identified by X-ray photoelectron spectroscopy (XPS) analysis (Fig. S3a†). Typically, Zn content decreases from 11.4 at% in ZIF-8 to 4.24 at% in ZIF-8@PDA and 1.99 at% in ZIF-8@mPDA, whereas the oxygen content increases dramatically from 3.20 at% in ZIF-8 to 20.0 at% in ZIF-8@PDA and 21.1 at% in ZIF-8@mPDA (Table S1†). Once ZIF-8 is coated with PDA, the elemental composition of ZIF-8 (zinc, nitrogen, and carbon) becomes substantially masked by that of PDA layer (oxygen, nitrogen, and carbon) because the thickness of PDA (∼25 nm) exceeds the detection depth of XPS (<10 nm). The nitrogen content also decreases from 26.3 at% in ZIF-8 to 8.96 and 7.41 at% in ZIF-8@PDA and ZIF-8@mPDA, respectively, due to the same reason (Table S1†). The ZIF-8 core is intact even after the PDA coating, therefore, forming a typical core–shell structure (Fig. 1c and d). The X-ray diffraction patterns of ZIF-8 in ZIF-8@PDA and ZIF-8@mPDA further confirm the presence of intact ZIF-8 in the core (Fig. S3b†). Fourier transform infrared spectra (FTIR) of ZIF-8, ZIF-8@PDA and ZIF-8@mPDA were measured to investigate their surface chemical bonding. FTIR spectra show C–O stretching vibrations at two regions (1275–1200 cm^−1^ and 1124–1087 cm^−1^), and C=O stretching vibration at 1760–1610 cm^−1^ in both types of PDA coated ZIF-8. These peaks originate from the catechol/quinone group of PDA (Fig. S3c and d†).

After the thermal annealing at 950 °C, ZIF-8, ZIF-8@PDA and ZIF-8@mPDA were successfully converted to microporous carbon (MPC), hollow microporous carbon (HMC) and hierarchically porous carbons (HPC), respectively (Fig. 1a). According to TEM images, ZIF-8 is observed to undergo significant shrinkage to ∼150 nm after its conversion to MPC (Fig. 1e and S1d†).^6^ This is due to the evaporation of Zn contents and collapse of some micropores at such high temperatures. ZIF-8@PDA and ZIF-8@mPDA, however, still maintain their particle size even after pyrolysis because their rigid PDA shells can effectively resist structural shrinkage (Fig. 1f, g, S1e and f†).

Interestingly, ZIF-8 undergoes a complete disintegration to form an obvious hollow cavity (macropore) in HMC (Fig. 1f). This is due to the strong outward interfacial force exerted on ZIF-8 by the rigid PDA shell.^44^ As the PDA shell undergoes decomposition at relatively low pyrolysis temperatures (<200 °C), it gains an increasing level of rigidity even from the initial stage of pyrolysis while ZIF-8 core slowly decomposes to acquire carbonaceous properties at higher temperature (∼600 °C). Their different decomposition rates consequently lead to uneven development of structural rigidity, thus giving rise to the disintegration of ZIF-8 and central hollowness through Kirkendall effect. In ZIF-8@mPDA, on the other hand, ZIF-8 only partially disintegrates and remains as porous inner shell within the mesoporous outer shell after pyrolysis, as indicated by the yellow-dotted circle in Fig. 1g (Fig. S4†).^20^

To better understand the carbonization process of ZIF-8@mPDA to form such unique nanoarchitecture, thermal gravimetrical analysis (TGA) was conducted with F127, ZIF-8, ZIF-8@PDA and ZIF-8@mPDA (Fig. S5a†). In the first stage (25 to 300 °C) of TGA, almost no weight loss is observed from ZIF-8 (∼1.5%), whereas there is a substantial weight loss from ZIF-8@PDA and ZIF-8@mPDA (∼10%). The initial weight loss of both ZIF-8@PDA and ZIF-8@mPDA can be attributed to the decomposition of PDA. In the subsequent stage (300 to 500 °C), ZIF-8 still maintains most of its initial weight, but ZIF-8@PDA and ZIF-8@mPDA continue to lose their weight at increased rates. The increased rates of thermal decomposition of ZIF-8@PDA and ZIF-8@mPDA at this stage are largely due to the disintegration of the ZIF-8 core by Kirkendall effect. As the remaining F127 in ZIF-8@mPDA fully decompose between 350 and 400 °C, the mesopores in the outer shell become more conspicuous (Fig. S5a and c†). In the last stage (500 to 900 °C), ZIF-8 undergoes a significant weight loss, and its weight loss reaches 10% at ∼587 °C. Such trend of ZIF-8 corresponds well with the previous literature data stating that ZIF-8 remains intact up to ∼550 °C, and its organic linkers thermally decompose and start to carbonize at ∼600 °C.^45,46^ As compared to ZIF-8, however, the rate of thermal decomposition is much more attenuated for both ZIF-8@PDA and ZIF-8@mPDA because their ZIF-8 core is already partially disintegrated in the previous stages by Kirkendall effect.

To further discuss the carbonization process of ZIF-8@mPDA, it was carbonized at specific temperatures (360, 580, 650 and 950 °C) marked by the TGA, then characterized by XRD and TEM analyses (Fig. S5b–e†). After annealing at 360 °C, the crystalline phase of ZIF-8 is still observable which is assignable to the ZIF-8 yolk in the TEM image (Fig. S5b and c†). This indicates that the disintegration of ZIF-8 core in ZIF-8@mPDA occurs from its peripheral interface with the mPDA shell. On the contrary, the disintegration of ZIF-8 core in ZIF-8@PDA begins from the center according to the previous report.^44^ The differing feature of the initial ZIF-8 core disintegration in ZIF-8@mPDA and ZIF-8@PDA is largely attributed to the distribution of stress induced on ZIF-8. Due to the presence of mesostructures, the mPDA shell exerts uneven stress on ZIF-8. Consequently, the initial disintegration of ZIF-8 happens away from the center. The PDA, however, exerts even stress on ZIF-8, hence inducing the disintegration of ZIF-8 from the center.^20,44^

As the annealing temperature increases to 580 °C, most diffraction peaks of ZIF-8 disappear while the peaks for ZnO appear due to unavoidable oxidation of metallic zinc in the sample at the contact to air atmosphere (Fig. S5b†).^40^ The TEM image also demonstrates highly decomposed yolk with numerous ZnO nanoparticles (Fig. S5d†). Upon annealing at 650 °C, the crystalline peaks of ZIF-8 and ZnO are no longer observed from the XRD spectrum, and a broad carbon peak at ∼26° appears (Fig. S5b†). The absence of ZnO peak is mainly due to the evaporation of majority of Zn species from the material, thus leaving only a negligible amount of Zn for oxidation in the air. At higher annealing temperature of 950 °C, further carbonization occurs and the two broad carbon peaks at 26° and 45° appear at higher intensity (Fig. S5b†). The mesoporous outer carbon shell and the porous inner shell are connected by the carbon scaffolds in HPC (Fig. S4 and S5e†).^20^

MPC, HMC and HPC clearly show the appearance of two broad carbon peaks and the disappearance of characteristic peaks of ZIF-8 in their XRD spectra, indicating the successful thermal conversion of ZIF-8, ZIF-8@PDA and ZIF-8@mPDA into their respective porous carbon materials (Fig. S6a†). Raman spectra of MPC, HMC and HPC show obvious D and G bands at ∼1355 cm^−1^ and ∼1585 cm^−1^, respectively (Fig. S6b†). It is generally claimed that D band represents disordered parts of the carbon structure while G band represents graphitized carbon structure.^6^ Therefore, the degree of graphitization can be inferred by the intensity ratio between D and G bands (*I*_D_/*I*_G_). It is found that MPC has lower *I*_D_/*I*_G_ value (0.94) than both HMC (*I*_D_/*I*_G_ = 0.97) and HPC (*I*_D_/*I*_G_ = 0.99), indicating its higher graphitization level. Based on XPS analysis, MPC, HMC and HPC are identified as nitrogen-doped carbons with high carbon contents of 88.0, 89.4 and 86.0 at% and nitrogen contents of 5.88, 4.65 and 4.89 at%, respectively (Fig. S6c, Table S2†). High-resolution XPS (HRXPS) spectra of C 1s reveal that a large proportion of carbon atoms are bonded to nitrogen in MPC, HMC and HPC (Fig. S6d, Table S3†). Specifically, different types of carbon-bonded nitrogen atoms can be classified into pyridinic, pyrrolic or graphitic nitrogen depending on their bonding configuration with neighboring carbon atoms. As pyridinic and graphitic nitrogen are known to contribute to ORR catalytic activity of carbon materials, HRXPS spectra of N 1s for the three carbon samples were investigated.^2,20^ Typically, the level of pyridinic and graphitic nitrogen increases in the order of HMC (3.47 at%) < HPC (3.65 at%) < MPC (4.20 at%) though the difference between the samples is rather marginal (Fig. 1h and S6e and Table S4†).

Nitrogen adsorption/desorption isotherms demonstrate a marked influence of each nanoarchitecture on the specific surface area (*S*_BET_) and pore size distribution. MPC has the highest *S*_BET_ of 889 m^2^ g^−1^ due to its abundant micropores comprising of 54.4% of its total pore volume (Fig. 1i and j and Table S5†).^6,35^ On the contrary, HMC and HPC demonstrate lower *S*_BET_ of 696 and 721 m^2^ g^−1^, respectively (Fig. 1j and Table S5†). Their nitrogen adsorption/desorption isotherms exhibit the mixture of type I and type IV isotherms and confirm the hierarchical porosity (Fig. 1i).^33^ In terms of pore size distribution, the ratio of micropore of HMC (34.4%) is nearly twice the value of HPC (17.5%) (Fig. 1j and Table S5†). This is due to the presence of well-defined mesopores on the outer shell of HPC. Pore size distribution of HPC clearly shows significantly higher volume of mesopores as compared to MPC and HMC (Fig. S7a†). According to SEM and TEM images, the mean pore size in the outer shell is 11.5 and 11.7 nm, respectively (Fig. S7b–e†). It is noteworthy that the mean pore size of the outer shell tends to increase as a larger amount of TMB is added to the synthesis. As the added TMB amount increases from 1.0 mL to 1.5 mL and to 2.5 mL, the mean pore size of the outer shell also increases from 7.65 nm to 9.46 nm and to 11.36 nm, respectively (Fig. S8†).

**Fig. 2:**
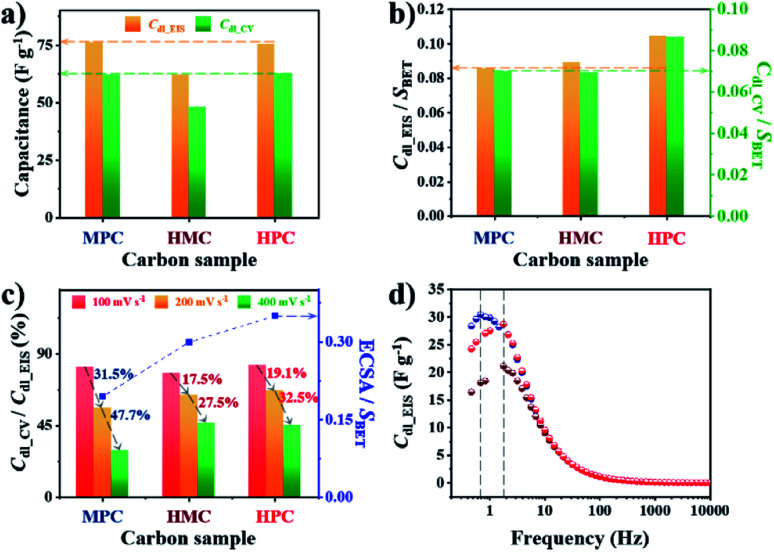
(a) Electric double layer capacitance calculated from EIS measured at OCP (*C*_dl_EIS_) and CV measured at 100 mV s^−1^ (*C*_dl_CV_) of MPC, HMC and HPC. (b) *C*_dl_EIS_ and *C*_dl_CV_ of MPC, HMC and HPC normalized by their *S*_BET_. (c) The ratio between *C*_dl_CV_ and *C*_dl_EIS_ at high scan rates (100, 200 and 400 mV s^−1^) and electrochemically active surface area (ECSA) normalized by *S*_BET_ of MPC, HMC and HPC. (d) Imaginary part of complex capacitance (*C*_im_) at OCP.

To investigate the effect of nanoarchitectures and the role of each class of nanopore, we conducted in-depth electrochemical analysis with electrochemical impedance spectroscopy (EIS). In this study, we prepared electrodes with small catalyst loading amount (0.2 mg cm^−2^) because the increase in electrode thickness by large catalyst loading amount can significantly limit both electron and mass transfer.^47^ Use of small catalyst loading amount also ensures the complete use of electrochemically wettable and accessible surface area of porous carbon materials. Prior to the electrochemical measurement, all electrodes were subjected to initial activation to achieve full wetting of the surface of the carbon materials, hence minimizing the effect of initial contact wettability (Fig. S9†). Next, the electric double layer capacitance (*C*_dl_) was calculated from EIS and cyclic voltammetry (CV) to obtain *C*_dl_EIS_ and *C*_dl_CV_, respectively. Specifically, *C*_dl_EIS_ represents electrochemical wettable surface area (EWSA) while *C*_dl_CV_ represents electrochemical active surface area (ECSA). The comparison of *C*_dl_EIS_ and *C*_dl_CV_ values of MPC, HMC and HPC can help to quantitatively evaluate the effect of their unique porous structures on both EWSA and ECSA. Interestingly, both MPC and HPC show similar *C*_dl_EIS_ and *C*_dl_CV_ values despite MPC having much higher physical surface area (*S*_BET_) than HPC (Fig. 2a, Table S5†). Such high *C*_dl_ values of HPC are largely due to the presence of mesopores which can effectively alleviate the overlapping of electric double layer occurring in small micropores. As a result, the unique nanoarchitecture of HPC helps to reduce the portion of its physical surface area that is electrochemically redundant/inactive.^48^ For MPC and HMC, which are largely deprived of mesopores, their EWSA and ECSA are linearly related to the physical surface area. This is further proven by their similar values of *S*_BET_ normalized *C*_dl_EIS_ and *C*_dl_CV_ (*C*_dl_EIS_/*S*_BET_ and *C*_dl_CV_/*S*_BET_, respectively) (Fig. 2b). On the contrary, HPC still exhibits significantly greater values of *C*_dl_EIS_/*S*_BET_ and *C*_dl_CV_/*S*_BET_, therefore, confirming that mesopores contribute to maximize ECWA and ECSA. Next, *C*_dl_CV_ values of MPC, HMC and HPC obtained at high scan rates (100 and 200, 400 mV s^−1^) were normalized by their *C*_dl_EIS_ to understand the percentage of ECWA being used as ECSA at high scan rates. Unlike MPC, both HMC and HPC demonstrate much improved retention of *C*_dl_CV_/*C*_dl_EIS_ values at higher scan rates, indicating the potential benefit of macropore in the rate capability (Fig. 2c). To further verify this, we calculated ECSA using the slope of linear plot of anodic current obtained at −0.025 V from 5 to 400 mV s^−1^ (Fig. S10†). The calculated ECSA values were then normalized by *S*_BET_, and it reveals that HMC and HPC are more suitable for maintaining the rate capability at a greater level than MPC (Fig. 2c). Therefore, it can be concluded that the hollow nanoarchitecture (macropore) is crucial for retaining ECSA at high charge–discharge rates. The importance of macropore in rate capability is further demonstrated by the relaxation time constant (*τ*_0_). Lower *τ*_0_ value indicates that the sample can form electric double layer even from the higher frequency. Both HMC and HPC exhibit significantly lower *τ*_0_ value of 0.09 s than 0.23 s of MPC. Hollow nanoarchitecture of HMC and HPC, therefore, effectively reduces ion transport resistance by acting as a reservoir of reactants, hence contributing to the rate capability (Fig. 2d).

**Fig. 3:**
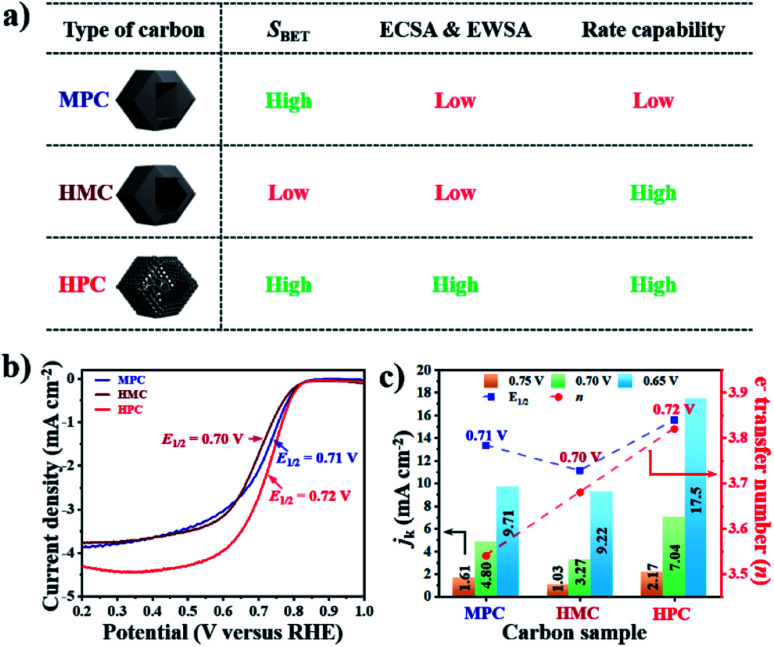
(a) Description of proposed ionic diffusion comparison between MPC, HMC, and HPC. (b) LSV curves and (c) kinetic current densities (*j*_k_, left *y*-axis), electron transfer number (right *y*-axis) and *E*_1/2_ (blue-dotted line) at 0.65, 0.70 and 0.75 V of MPC, HMC and HPC.

The summary of the effect of nanoarchitecture on ECWA, ECSA and rate capability is illustrated in Fig. 3a. ORR activity of MPC, HMC and HPC was then investigated to demonstrate our conclusion. From LSV curves, all samples show onset potential (*E*_onset_) of 0.82 V in O_2_-saturated alkaline electrolyte (Fig. 3b and Table S6†). HPC exhibits higher kinetic current density (*J*_k_) than both MPC and HMC between 0.8 and 0.2 V due to its high ECSA facilitating significantly efficient mass transport (Fig. 2). HPC steadily generates the highest *J*_k_ among the samples at all potential due to the significant benefit of its unique nanoarchitecture with trimodal porosity (Fig. 3c). Herein, we propose that the control of porosity and pore distribution based on the in-depth understanding of the specific role of each class of nanopore proposed in this study is very important in achieving desired electrochemical properties (*e.g.*, high ORR catalytic performance).^49,50^

**Fig. 4:**
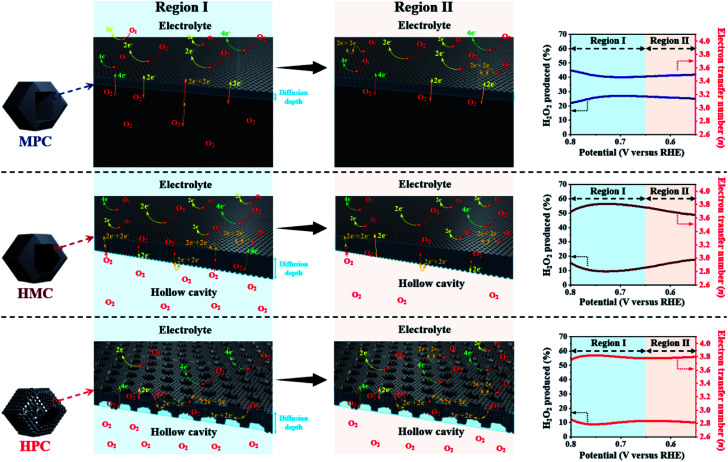
Graphical description of proposed effect of different nanoarchitectures (MPC, HMC and HPC) on overall electron transfer number calculated from RRDE method (4e^−^, 2e^−^ + 2e^−^ and 2e^−^ indicate four electron, double two electron and single two electron transfer pathways, respectively).

HPC also demonstrates the highest electron transfer number of 3.82 and the lowest H_2_O_2_ production of 8.80% at the potential range between 0.20 and 0.80 V, while MPC has the lowest electron transfer number of 3.54 and the highest H_2_O_2_ production of 23.0% at the same potential range. Such high percentage yield of H_2_O_2_ by MPC largely matches with most literature (Fig. 4 and Table S6†).^51–53^ As rotating ring disk electrode (RRDE) method largely relies on the reduction and oxidation currents generated by disk electrode and platinum ring electrode, respectively, it can potentially result in an overall electron transfer by single four-electron (4e^−^), double two-electron (2e^−^ + 2e^−^), and single two-electron (2e^−^) pathways. Consequently, we can attribute the difference in the electron transfer number between the samples to their distinct nanoarchitectures (Fig. 4). In region I (0.80 to 0.65 V), the diffusion of molecules in MPC is highly restricted to the small micropores near the exterior surface. H_2_O^−^ molecules generated by the single 2e^−^ pathway near the exterior surface, therefore, diffuse out from MPC (Fig. 4). On the contrary, the depth of diffusion of O_2_ molecules for ORR is expected to be much greater for HMC than MPC because the diffusion takes place in and out of the particle using both ends of its thin microporous shell (Fig. 4). Consequently, more ORR can take place deeper in HMC than MPC. Moreover, potential H_2_O^−^ generated and trapped in the hollow cavity stands higher chance to be further reduced to H_2_O through the second 2e^−^ pathway occurring while diffusing out from interior to exterior of HMC (Fig. 4). This contributes to HMC having a greater overall electron transfer number than MPC in the region I. HPC shows the highest ORR catalytic activity and overall electron transfer number in the region I as its hierarchical porosity, especially mesopores, can effectively facilitate 2e^−^ + 2e^−^ pathways. As the potential approaches towards region II (below 0.65 V), the overall *n* value of MPC remains rather steady because the consumption of O_2_ molecules is highly concentrated at the surface (Fig. 4). In the case of HMC, O_2_ and H_2_O^−^ in the hollow cavity of HMC and become less available by the continuous consumption. As a result, further consumption of O_2_ molecules occurs more likely at the exterior surface of HMC, hence making the second 2e^−^ pathway less frequent and decrease in the overall *n* value (Fig. 4). On the contrary, HPC does not exhibit much decrease in the overall *n* value even in the region II because the presence of mesopores allows efficient replenish of O_2_ molecules into the interior, thus keeping ORR to take place continuously at both ends of the thin micro-/mesoporous shell (Fig. 4). HPC can, therefore, efficiently facilitate 2e^−^ + 2e^−^ pathway even at high overpotential and exhibit highest overall electron transfer number among the carbon samples.

**Fig. 5:**
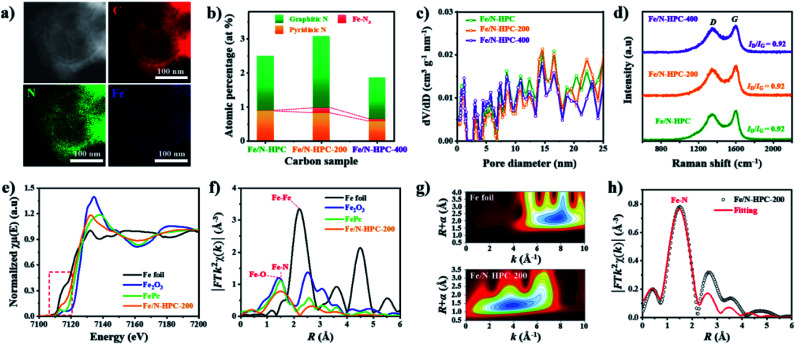
(a) STEM-EDS images of Fe/N–HPC-200. (b) Atomic percentage of different nitrogen species, (c) NLDFT pore size distributions and (d) Raman spectra of Fe/N–HPC, Fe/N–HPC-200 and Fe/N–HPC-400. (e) XANES and (f) FT-EXAFS curves of Fe/N–HPC-200 and references at Fe K-edge. (g) WT-EXAFS of Fe foil and Fe/N–HPC-200. (h) FT-EXAFS fitting curve of Fe/N–HPC-200.

Next, additional Fe dopants were introduced to HPC to obtain highly active Fe, N co-doped HPC. N content of HPC, however, is rather low while its O content is relatively high. This potentially renders HPC to stand a limited chance of forming Fe–N coordination bonding not only by the lack of N atoms but also by the abundance O atoms that can also react with Fe. For such reason, more nitrogen atoms are introduced to ZIF-8@mPDA by adding N-rich melamine molecules prior to Fe doping.^39–41^ Melamine molecules can copolymerize during DA polymerization, hence successfully increasing N content in the resulting PDA. Indeed, the N content increases from 7.41 at% in ZIF-8@mPDA to 7.97 at% in ZIF-8@mPDA-200 and to 8.94 at% in ZIF-8@mPDA-400 (where the number indicates the mass of melamine added in mg) as more melamine is added (Table S1†). With increased nitrogen content, the chance of forming coordination bonding between Fe and N to Fe–N_*x*_ in HPC is, therefore, expected to be higher. A series of Fe, N co-doped HPC (Fe/N–HPC) samples was prepared by acidic treatment of Fe nanoparticle loaded HPC (FeNP–HPC) (Fig. S11a†). After the acid treatment, the intensity of diffraction peaks from iron species becomes significantly weaker, indicating that most unstable Fe species are removed. Some iron oxides (Fe_2_O_3_), however, remain encapsulated by the carbon matrix even after the acid treatment because the formation of Fe_2_O_3_ is inevitable due to the oxygen-rich nature of PDA (Fig. S11b†). Fe/N–HPC prepared with varying amounts of Fe precursor were first compared for ORR electrocatalysis (Fig. S12†). It is found that 5 wt% of Fe precursor produces the most active ORR electrocatalyst, thus it is selected as the standard Fe precursor amount in this study (Fig. S12†). Following the same procedures, Fe/N–HPC-200 and Fe/N–HPC-400 were also prepared with ZIF-8@mPDA-200 and ZIF-8@mPDA-400, respectively. Fe/N–HPC, Fe/N–HPC-200 and Fe/N–HPC-400 show no notable morphological deviation, indicating that the presence of melamine during DA polymerization neither have significant influence on the formation of mesostructures nor cause abrupt morphological change during carbonization (Fig. 5a and S13a–d†). Elemental mapping of Fe/N–HPC-200 demonstrates that both Fe and N are uniformly distributed across the carbon matrix (Fig. 5a and S13e–i†). From the high-angle annular dark-field scanning transmission electron microscopy (HAADF-STEM) analysis, Fe single atoms are clearly observed in Fe/N–HPC-200 (Fig. S13j and k†). According to XPS analysis, Fe/N–HPC, Fe/N–HPC-200 and Fe/N–HPC-400 contain C, N, and Fe, demonstrating that they are successfully co-doped with Fe and N (Fig. S14a and b and Table S2†). As the same amount of Fe precursor (5 wt%) is used for doping, no significant difference in Fe content is identified between Fe/N–HPC (0.30 at% or 1.34 wt%), Fe/N–HPC-200 (0.29 at% or 1.30 wt%) and Fe/N–HPC-400 (0.30 at% or 1.34 wt%) based on XPS elemental analysis (Table S2†). However, HRXPS spectra for N 1*s* indicate that the level of Fe–N_*x*_ peak tends to increase upon the addition of melamine from Fe/N–HPC (0.01 at%) to Fe/N–HPC-400 (0.09 at%) and Fe/N–HPC-200 (0.15 at%) (Fig. 5b and S14c and Table S7†).^54–57^ It indicates that Fe/N–HPC-200 is the optimized ORR catalyst as it has the highest density of Fe–N_*x*_. Nitrogen adsorption/desorption isotherms of Fe/N–HPC, Fe/N–HPC-200 and Fe/N–HPC-400 show the mixture of type I and type IV isotherms with a wide pore size distribution in both micro- and mesopore ranges (Fig. 5c and S13l†). Based on the morphological observations and pore size distributions, we confirm that the structural effect on ORR electrocatalysis is largely eliminated among Fe/N–HPCs. The earlier investigation of different nanoarchitectures demonstrates that the surface area is not always the absolute parameter to determine the intrinsic ORR catalytic activity of the catalyst. The effect of small variations in *S*_BET_ among Fe/N–HPCs are, therefore, predicted to be marginal towards the difference in their ORR catalytic activities (Table S5†). After Fe doping, *I*_D_/*I*_G_ ratio of Fe–HPC, Fe–HPC-200 and Fe–HPC-400 are found to be 0.92 which is much lower than that of the pristine HPC (0.99), indicating the increased level of graphitization due to the presence of Fe during carbonization (Fig. 5d and S6b†). To further understand the ORR catalytic active sites at the atomic level, X-ray adsorption near-edge structure (XANES) and extended X-ray adsorption fine structure (EXAFS) of Fe/N–HPC-200 were measured. XANES curve of Fe/N–HPC-200 demonstrate higher pre-edge adsorption energy than that of Fe foil, implying that its Fe atoms carry positive charges (Fig. 5e).^18^ FT-EXAFS curve of Fe/N–HPC-200 shows the peak at about 1.5 Å, assignable to Fe–N coordination. This peak, however, is broadened to include Fe–O peak due to the presence of iron oxides in the sample. Fe/N–HPC-200 does not show Fe–Fe peak at about 2.2 Å, indicating that unstable Fe nanoparticles are successfully removed (Fig. 5f). To further elucidate the presence of atomically dispersed Fe atoms in Fe/N–HPC-200 and visualize both *k* and *R* spaces, wavelet transform (WT) of Fe K-edge EXAFS oscillations was obtained. From WT-EXAFS, a single maximum intensity is observed at about 8 Å^−1^ for Fe foil and 4 Å^−1^ for Fe/N–HPC-200, and they correspond to Fe–Fe and Fe–N bonds, respectively (Fig. 5g).^58^ It can be, therefore, concluded Fe/N–HPC-200 carries Fe atoms that are atomically dispersed and nitrogen-coordinated in its hierarchically porous nanoarchitecture. A least-squares EXAFS fitting was also carried out to obtain quantitative structural parameters of Fe in the Fe/N–HPC-200 (Fig. 5h). Although the XANES and FT-EXAFS analysis of Fe/N–HPC is similar in trend as that of Fe/N–HPC-200, its peak in FT-EXAFS is slightly negative-shifted closer to Fe–O bond as compared to that of Fe/N–HPC-200, indicating that Fe–O bond is more prone to form without introducing melamine as additional N sources (Fig. S15†). The EXAFS fitting parameters are listed in Table S8.†

**Fig. 6:**
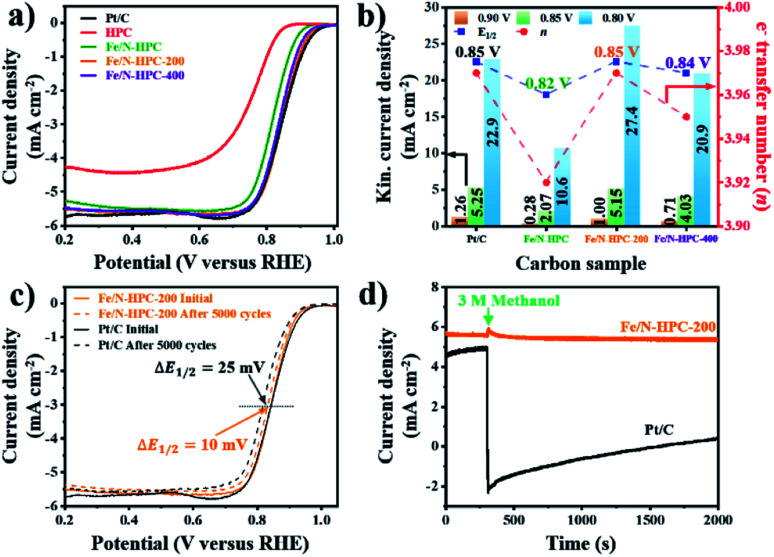
(a) LSV curves of Pt/C, HPC and Fe/N–HPC, Fe/N–HPC-200 and Fe/N–HPC-400. (b) Electron transfer number, *E*_1/2_ and kinetic current density of Pt/C, Fe/N–HPC, Fe/N–HPC-200 and Fe/N–HPC-400. (c) LSV curves of Pt/C and Fe/N–HPC-200 before and after the accelerated durability test (ADT). (d) Chronoamperometric response of Pt/C and Fe/N–HPC-200 with 3 M methanol at 0.65 V and 1600 rpm. All data are obtained in O_2_-saturated 0.1 M KOH electrolyte.

In terms of ORR performance, all Fe/N-doped carbons show highly improved catalytic activity as compared to pristine HPC with significantly more positive *E*_onset_ as well as higher electron transfer number and lower H_2_O_2_ yield (Fig. 6a and S16a and Table S6†). Nevertheless, the ORR electrocatalytic activity of Fe/N–HPC is still behind that of state-of-the-art ORR electrocatalyst, Pt/C, by 50 mV for *E*_onset_ and 30 mV for *E*_1/2_ (Fig. 6a and Table S6†). Moreover, Fe/N–HPC cannot reach high electron transfer number of Pt/C (3.97) while producing almost three times more H_2_O_2_ than Pt/C (1.70%) (Fig. S16a and Table S6†). Surprisingly, LSV curves of Fe/N–HPC-200 and Fe/N–HPC-400 demonstrate significant improvement in *E*_onset_ to 0.96 and 0.95 V, respectively, which are highly comparable to that of Pt/C (0.97 V) (Fig. 6a and Table S6†). Furthermore, they also exhibit much more positive *E*_1/2_ of 0.85 V and 0.84 V, respectively, as compared to Fe/N–HPC (0.82 V), and the value of Fe/N–HPC-200 is observed to be identical to that of Pt/C (Fig. 6b and Table S6†). The calculation of electron transfer number from the RRDE method demonstrates near four-electron ORR pathway by Fe/N–HPC-200 having the same electron transfer number of 3.97 as Pt/C, and Fe/N–HPC-400 having slightly lower value of 3.95 over the potential range between 0.20 and 0.80 V (Fig. 6b and Table S6†). Koutecky–Levich (K–L) plots of Fe/N–HPC-200 and Fe/N–HPC-400 also show high linearity and their electron transfer numbers are found to be close to the ideal four-electron ORR pathway in both acidic and alkaline electrolytes (Fig. S16b–e and Table S6†). The *j*_k_ value of Fe/N-doped carbons seems to be largely influenced by the density of Fe–N_*x*_ sites as it increases from Fe/N–HPC to Fe/N–HPC-400 and to Fe/N–HPC-200, exhibiting the identical increasing trend for Fe–N_*x*_ density among them (Fig. 5b and 6b). It is generally accepted that Fe–N_*x*_ site offers energetically favorable adsorption site for oxygen to promote superior ORR catalytic activity although its mechanism is yet to be fully elucidated.^59–61^ According to HRXPS for N 1s of Fe/N–HPC, Fe–N_*x*_ peak is present only in a negligible level (0.01 at%) although Fe 2p peak is clearly seen (Fig. 5b, S14c and d†). It therefore suggests that most N and Fe are present unbonded to each other thus lacking Fe–N_*x*_ coordination in Fe/N–HPC. As N and Fe are known to have significantly lower catalytic activity than Fe–N_*x*_, it is likely that their separate catalytic effects largely limit the ORR catalytic activity of Fe/N–HPC.^60,61^ Interestingly, the rate of increase in *j*_k_ for Fe/N–HPC-200 is greater than that for Pt/C at more negative potentials, thus demonstrating its increasingly higher ORR catalytic activity in O_2_-saturated 0.1 M KOH electrolyte (Fig. 6b). The ORR catalytic activity of Fe/N–HPC-200 is especially notable among the previous literature with consideration of its small catalyst loading amount (Table S9†). In O_2_-saturated 0.1 M HClO_4_ electrolyte, however, the ORR catalytic activity of Fe/N–HPC-200 and Fe/N–HPC-400 becomes lower than that of Pt/C because it is thermodynamically more challenging to achieve a lower overpotential for the ORR as the electrolyte pH decreases (Fig. S17†). The CV curves of Fe/N–HPC-200 and Fe/N–HPC-400 show highly selective ORR catalytic activity in both O_2_-saturated 0.1 M KOH and 0.1 M HClO_4_ electrolytes unlike Pt/C undergoing side reactions (Fig. S17b and c†).

Apart from the catalytic activity, long-term stability and methanol tolerance are highly important features to be considered for ORR electrocatalyst. The long-term stability of Fe/N–HPC-200, Fe/N–HPC-400 and Pt/C catalysts was assessed by the following accelerated durability test (ADT) protocol. In ADT, 5000 cycles of cyclic voltammetry (CV) were conducted over the potential range between 0.6 and 1.0 V in O_2_-saturated 0.1 M KOH, and LSV curves at a rotation speed of 1600 rpm were compared before and after 5000 consecutive cycles of CV. In 0.1 M KOH, Fe/N–HPC-200 and Fe/N–HPC-400 exhibit exceptional durability with only a slight negative shift of *E*_1/2_ by 10 mV and 9 mV, respectively, after 5000 cycles while more than twice potential decay (25 mV) is observed for Pt/C (Fig. 6c, S18a and b†). The difference in long-term stability is more conspicuous in O_2_-saturated 0.1 M HClO_4_ electrolyte as the change in *E*_1/2_ is observed to be 30 mV and 40 mV for Fe/N–HPC-200 and Fe/N–HPC-400, respectively, whereas it reaches 130 mV for Pt/C (Fig. S17d and e†). The tolerance towards methanol poisoning was also assessed in O_2_-saturated 0.1 M KOH. In the presence of 3 M methanol, the CV curves of Pt/C show loss of catalytic specificity towards ORR (Fig. S18c†). On the other hand, the CV curves of Fe/N–HPC-200 and Fe/N–HPC-400 demonstrate that they can largely maintain their catalytic specificity towards ORR even in the presence of methanol (Fig. S18c†). Furthermore, their LSV curves at 1600 rpm show only slight decrease in *E*_1/2_ by 8 and 13 mV, respectively, while that of Pt/C exhibits a significant methanol oxidation peak at ∼0.9 V (Fig. S18d–f†). Chronoamperometric response of Fe/N–HPC-200 also shows very stable current density with quick recovery from a slight disruption at the time of methanol addition. On the contrary, a dramatic decrease in current density is observed for Pt/C as soon as methanol had been added (Fig. 6d).

## Conclusions

The importance of strategical designing of nanoarchitectures to improve electrochemical properties is demonstrated with three types of N-doped ORR electrocatalysts with different porosity. The electrochemical comparison of MPC, HMC, and HPC reveals the role of each class of nanopore towards specific electrochemical properties and the overall ORR catalytic activity as follows: (1) micropores contribute towards physical surface area while severely restricting electrochemical wettability and accessibility. (2) Mesopores effectively increase electrochemical wettability and accessibility and maintain physical surface area to a large extent. (3) Macropores contribute towards rate capability and mass transport but significantly sacrifice the physical surface area. HPC can be selected as the model porous carbon scaffold for the subsequent Fe doping. The density of Fe–N_*x*_ sites is elevated by eliminating potential limiting factors, in this case the N content, to achieve notable increase in the ORR catalytic activity. We, therefore, conclude that the strategic consideration of structural and doping effects is paramount in achieving advanced catalyst (or electrode) materials for energy conversion/storage applications.

## Data availability

The data that support the findings of this study are available from the corresponding authors upon reasonable request.

## Author contributions

M. K., J. T. and Y. Y. designed the research; M. K., K. L. F., J. F. S. F., X. X. and H. L. conducted the research; M. K., K. L. F., J. F. S. F., D. V. G., J. N., J. K., H. N., J. T., and Y. Y. analyzed the data; M. K., H. N., J. T. and Y. Y. wrote the paper.

## Conflicts of interest

There are no conflicts to declare.

## Supplementary Material

SC-013-D2SC02726G-s001
