# Supplementary material for: Strategic design of Fe and N co-doped hierarchically porous carbon as superior ORR catalyst: from the perspective of nanoarchitectonics
Source: Chem Sci. 2022 Sep 1;13(36):10836–45. doi: 10.1039/d2sc02726g (PMC9491178; doi:10.1039/d2sc02726g)
Supplement: SC-013-D2SC02726G-s001 [file SC-013-D2SC02726G-s001.pdf]

Electronic Supplementary Material (ESI) for Chemical Science.
This journal is © The Royal Society of Chemistry 2022

Electronic Supporting Information for

Strategic Design of Fe and N Co-Doped Hierarchically Porous Carbon as Superior ORR Catalyst: From The Perspective of Nanoarchitectonics

Minjun Kim[a], Konstantin L. Firestein[b], Joseph F.S. Fernando[b], Xingtao Xu[c], Hyunsoo Lim[d], Dmitri V. Golberg[b,c], Jongbeom Na[a,e], Jihyun Kim[f], Hiroki Nara[c]*, Jing Tang[g]* and Yusuke Yamauchi[a,c]*

a. Australian Institute for Bioengineering and Nanotechnology (AIBN) and School of Chemical Engineering, The University of Queensland, Brisbane, Queensland 4072, Australia

b. Centre for Materials Science and School of Chemistry and Physics, Queensland University of Technology (QUT), 2 George Street, Brisbane, Queensland, 4000, Australia

c. International Center for Materials Nanoarchitechtonics (WPI-MANA), National Institute for Materials Science (NIMS), 1-1 Namiki, Tsukuba, Ibaraki 305-0044, Japan

d. New & Renewable Energy Research Center, Korea Electronics Technology Institute (KETI), 25, Saenari-ro, Bundang-gu, Seongnam-si, Gyeonggi-do 13509, Republic of Korea

e. Materials Architecturing Research Center, Korea Institute of Science and Technology, 5 Hwarang-ro 14-gil, Seongbuk-gu, Seoul 02792, Republic of Korea

f. Solar Energy R&D Department, Green Energy Institute, Mokpo, Jeollanamdo 58656, Republic of Korea

g. School of Chemistry and Molecular Engineering, Shanghai Key Laboratory of Green Chemistry and Chemical Processes, East China Normal University, Shanghai, 200062 China

* Corresponding authors at: National Institute for Materials Science (NIMS), 1-1 Namiki, Tsukuba, Ibaraki 305-0044, Japan (H. Nara); East China Normal University, Shanghai, 200062 China (J. Tang); The University of Queensland, Brisbane, Queensland 4072, Australia (Y. Yamauchi)

E-mail addresses: nara.hiroki@nims.go.jp (H. Nara), jingtang@chem.ecnu.edu.cn (J. Tang), y.yamauchi@uq.edu.au (Y. Yamauchi)

EXPERIMENTAL SECTION

Synthesis of ZIF-8

In a typical synthesis of zeolitic imidazolate framework-8 (ZIF-8), zinc acetate tetrahydrate (18.7 g) and 2-methylimidazole (56.0 g) were dissolved separately into 250 mL of deionized water. Next, the two solutions were mixed and stirred for 10 min. The resulting mixture was aged at room temperature without stirring for 24 h. White precipitates formed on the bottom were thoroughly washed with deionized water and ethanol several times and dried at 60 °C to obtain ZIF-8.

Synthesis of ZIF-8@PDA

In a typical synthesis of nonporous polydopamine coated ZIF-8 (ZIF-8@PDA), 100 mg ZIF-8 and 100 mg dopamine hydrochloric acid (Sigma-Aldrich) were dissolved in 50 mL of deionized water and stirred at 400 rpm. After stirring for 10 min, 130 mg Tris buffer (Bio-Rad) was added, and the mixture was stirred at 400 rpm for 6 h. The final products were washed thoroughly with deionized water and ethanol several times and dried at 60 °C to obtain ZIF-8@PDA.

Synthesis of ZIF-8@mPDA

In a typical synthesis of mesochanneled polydopamine coated ZIF-8 (ZIF-8@mPDA), 1.00 g Pluronic F127 (Sigma-Aldrich) and 200 mg dopamine hydrochloric acid (Sigma-Aldrich) were dissolved in 100 mL of deionized water/ethanol (1:1) solution and stirred. Once fully dissolved, 2.50 mL of 1,3,5-trimethlybenzene (TMB, Sigma-Aldrich) was added dropwise and the mixture was stirred for 2 h at 150 rpm. 100 mg ZIF-8 was then added and mixed for 30 min. After 30 min, 130 mg Tris buffer (Bio-Rad) was added, and the mixture was left stirring at 150 rpm for 24 h. The final products were washed thoroughly with deionized water and ethanol several times and dried at 60 °C to obtain ZIF-8@mPDA. The synthetic method of ZIF-8@mPDA-*x* (where *x* indicates the weight of melamine added in mg) follows that of ZIF-8@mPDA, except that *x* mg (typically 200 and 400 mg in this study) of melamine (Sigma-Aldrich) were added into 100 mL of deionized water/ethanol (1:1) solution along with Pluronic F127 and dopamine hydrochloric acid.

Synthesis of Fe/N-HPC and Fe/N-HPC-*x*

Fe, N co-doped hierarchically porous carbons (denoted as Fe/N-HPC) was synthesized by introducing Fe precursors to carbons derived from ZIF-8@mPDA. In a typical synthesis of Fe/N-HPC, ZIF-8@mPDA were pyrolyzed at 650 °C for 2 h under a N_2 atmosphere with a heating rate of 5 °C min^{-1} to obtain pre-HPC. 50.0 mg of pre-HPC and 18.1 mg of $Fe(NO_3)_3·9H_2O$ (Sigma-Aldrich) were mixed in 25 mL of deionized water at 500 rpm for 2 h. The mixed solution was then dried at 60 °C. The dried pre-HPC with Fe precursors were pyrolyzed at 950 °C for 2 h under a N_2 atmosphere with a heating rate of 5 °C min^{-1}. As-prepared Fe nanoparticle-loaded HPC (FeNP-HPC) was washed with 3 M H_2SO_4 at 100 °C for 24 h to remove aggregated Fe nanoparticles. After

24 h, the carbon samples were collected and washed with deionized water until pH becomes neutral, and dried at 60 °C. The carbon samples were pyrolyzed at 950 °C for 2 h under a N_2 atmosphere with a heating rate of 5 °C min^{-1} to obtain Fe/N-HPC. The same synthetic procedure was applied to synthesize Fe/N-HPC-*x* from ZIF-8@mPDA-*x*.

Materials Characterization

Field emission-scanning electron microscopy (FE-SEM) was performed at a JEOL JSM-7800F microscope at 2.0 kV. Transmission electron microscopy (TEM) and scanning TEM (STEM) were carried out at 120 kV with a TEM microscope HT7700 and using a 200 kV double-aberration-corrected JEM-200ARM "NeoARM" microscope. Powder X-ray diffraction (PXRD, D8 Advance, Bruker) was used to study crystal structures of materials with Cu-Kα radiation at 40 kV and 40 mA. The elemental composition and the electronic structure were investigated by X-ray photoelectron spectroscopy (XPS) using mono-Al Kα (1486.6 eV) X-rays. Thermal gravimetric analysis (TGA) was conducted with METTLER TOLEDO TGA/DSC 1 STAR e System from room temperature to 900 °C with a heating rate of 5 °C/min. The porosity and specific surface area of the samples were measured by BELSORP Mini X surface area and pore size analyzer. Fe K-edge X-ray absorption spectra (XAFS) were measured on 1W1B beamline of the Beijing Synchrotron Radiation Facility (BSRF) in fluorescence mode at a room temperature with Si (111) double-crystal monochromator. The storage ring of BSRF was conducted at 2.5 GeV with a maximum current of 250 mA in decay mode. After conducting the standard procedures with the ATHENA program, the raw data of XAFS was background-subtracted, normalized, and Fourier transformed. Least-squares curve fitting of the extended X-ray absorption fine structure χ(k) data were analyzed by using the ARTEMIS program. All fits were carried out in the R space with k-weight of 3.

Electrochemical Measurement

All electrochemical measurements were carried out using CHI760 (CH Instruments) under three electrode systems. The counter electrode and reference electrode used were a Pt wire and Hg/HgO electrode saturated with 1 M NaOH, respectively. The rotating ring disk electrode (Pt ring with glass carbon disk RRDE, ALS) was used as the working electrode, and the area was 0.126 cm^2. The catalyst ink was prepared using a mixture of catalyst and Nafion ionomer (15 wt%, Sigma-Aldrich) in 1:2 deionized water/2-isopropanol (Sigma-Aldrich). The catalyst loading of carbon materials in alkaline and acid electrolyte measurements was 0.2 mg cm^{-2}. To compare the activity, 20 wt% commercial Pt electrocatalyst (Alfa Aesar) was used. The Pt loading was 40 μg_{Pt} cm^{-2}. The electrochemical measurements were conducted in O_2/N_2-saturated 0.1 M KOH and 0.1 M $HClO_4$ electrolytes. Cyclic voltammetry (CV) was performed at various scan rates from 5 to 400 mV s^{-1}, and linear sweep voltammetry (LSV) was performed at a rotating speed of 1600 rpm with a scan rate of 10 mV s^{-1}. The LSV measurements were also conducted at different rotating speed from 400 to 2500 rpm. Electrochemical impedance spectroscopy (EIS) was performed at open circuit potential without rotating with

an amplitude of 10 mV between 100 kHz and 50 mHz. Accelerated durability tests (ADT) were carried out by cycling the catalyst at a potential window of 0.6 to 1.0 V with a scan rate of 50 mV s^{-1} in O_2-saturated 0.1 M KOH and 0.1 M $HClO_4$ electrolytes for 5000 cycles. LSV curves at a rotating speed of 1600 rpm at a scan rate of 10 mV s^{-1} were obtained before and after the ADT. Methanol tolerance of catalyst was observed by *i-t* chronoamperometric curve in O_2-saturated 0.1 M KOH electrolyte at a rotating speed of 1600 rpm with 3 M methanol added after 300 s. The non-Faradaic capacitive effect was compensated by subtracting the current measured in N_2 saturated condition.

All the potentials in the measurements were converted to potentials *vs*. the reversible hydrogen electrode (RHE) according to the following equation:

$$E_{RHE} = E_{Hg/HgO} + (0.0591 \times pH) + 0.118$$

Electron transfer number (*n*) was calculated according to the Koutecky-Levich equation:

$$\frac{1}{j} = \frac{1}{j_L} + \frac{1}{j_K} = \frac{1}{Bw^{1/2}} + \frac{1}{j_K}$$

$$B = 0.62nFC_0D^{2/3}{}_0v^{-1/6}$$

where w is the angular velocity, j is the measured current density, j_K and j_L are the kinetic and diffusion-limiting current densities, respectively, n is the number of transferred electrons, F is the Faraday constant (96485 C mol^{-1}), C_0 is the bulk concentration of O_2 (1.2×10^{-6} mol cm^{-3}), D_0 is the diffusion coefficient of O_2 (1.9×10^{-5} cm^2 s^{-1}), and v is the kinematic viscosity of the electrolyte (0.01 cm^2 s^{-1}).

The value of *n* from RRDE method was calculated by the following equation:

$$n = 4 \times \frac{i_d}{i_d + \frac{i_r}{N_c}}$$

Percentage yield of H_2O_2 was also calculated by the following equation:

$$\%H_2O_2 = 200 \times \frac{\frac{i_r}{N_c}}{i_d + \frac{i_r}{N_c}}$$

where N_c is the collection efficiency of RRDE (in our case: 0.37), which is related by the distance from the disk electrode to ring electrode, and i_d and i_r are the disk and ring currents, respectively.

Double layer capacitance by CV (C_{dl_CV}) was calculated by the following equation:

$$C_{dl\_CV} = \frac{1}{2 \times v \times m}\int Idt = \frac{1}{2 \times v \times m \times \Delta V}\int IdV$$

where v and ΔV are the scan rate (V s^{-1}) and the scan range (V) of CV, respectively, m is the mass of catalyst (g).

Double layer capacitance by EIS (C_{dl_EIS}) was calculated from complex capacitance, which is applicable for multiple pore systems.[1] The complex capacitance $C(\omega)$ was represented as following:

$$C(\omega) = \frac{1}{j\omega Z(\omega)}$$

where ω is angular frequency (rad s^{-1}) and $Z(\omega)$ is complex impedance as a function of angular frequency.

Note: To calculate C_{dl_EIS}, the raw data from low frequency region of EIS are used. The equation 'C(ω)=1/jωZ(ω) ' can be represented to the following equivalent circuit:

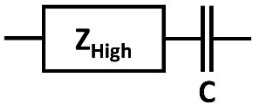

The equivalent circuit above is composed of the impedance at the high frequency region, which include electric and ionic resistances, and capacitance in series. In the low frequency region, however, the impedance of Z_{High} is constant, so that C_{dl_EIS} can be calculated simply from the raw data in the low frequency region.

The real and imaginary parts of $C(\omega)$ were derived from the following equations:

$$C(\omega) = C'(\omega) - jC''(\omega)$$

$$Z(\omega) = Z'(\omega) - jZ"(\omega)$$

$$C'(\omega) = -\frac{Z"(\omega)}{\omega|Z"(\omega)|^2},\ C"(\omega) = -\frac{Z'(\omega)}{\omega|Z"(\omega)|^2}$$

The total capacitance (C_0), that is C_{dl_EIS}, can be estimated theoretically from a cross point of $C'(\omega)$ with x-axis at extremely low angular frequency. In this study, however, it was difficult to obtain the value at such extremely low angular frequency due to the limitations of instrument and measuring time. Therefore, the C_0 was generally estimated from an imaginary capacitance plot against angular frequency, integrating the peak area (A). The relationship between the A and the C_0 was represented as following:

$$A = -\int_{-\infty}^{\infty} C"(\omega) d\log f = 0.682 C_0$$

where the factor 0.682 was obtained by a numerical integration of $C''(\omega)$ using the Kronig-Kramers transform.[2-4] Furthermore, we estimated the C_0 by the following formula reported by Lee et al. due to the limitations of instrument and measuring time.[1]

$$C_0 = -\frac{2}{0.682}\int_{f_0}^{10^5} C"(\omega) d\log f$$

where f_0 represents an angular frequency at the maximum of $C''(\omega)$ and $\int_{f_0}^{10^5} C"(\omega) d\log f$ represents the half area of A in this experiment. This calculation was based on the hypothesis that $C''(\omega)$ against log f is symmetric about f_0. However, in this study, f_0 that is point of symmetry, was unclear, so that f_0 was fixed to be 1 Hz, and the $C''(\omega)$ against log f lower than f_0 was actually larger (**Figure 2d**). Therefore, C_{dl_EIS} would be calculated smaller than the actual value.

The relaxation time constant (τ_0) was calculated by the following equation:

$$\tau_0 = \frac{1}{2\pi \times f_0}$$

where f_0 represents an angular frequency at the maximum total capacitance (C_0).

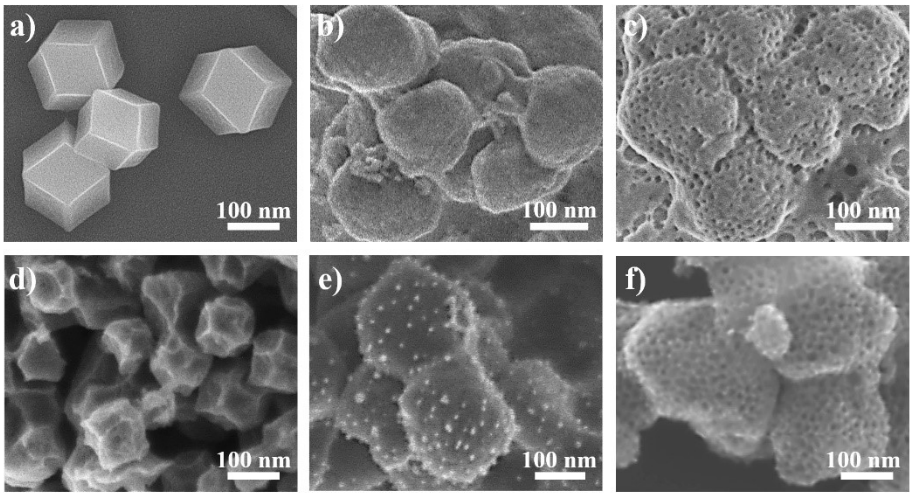

Figure S1. Field-emission scanning electron microscopy (FE-SEM) images of (a) ZIF-8, (b) ZIF-8@PDA, (c) ZIF-8@mPDA, (d) MPC, (e) HMC and (f) HPC.

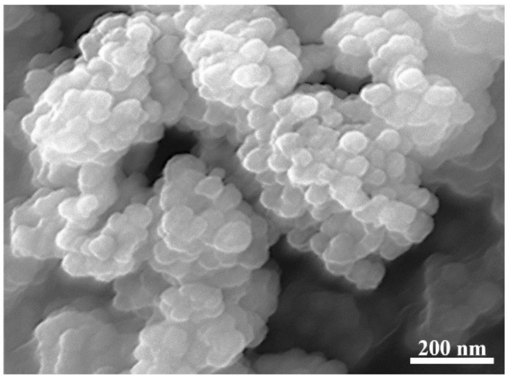

Figure S2. FE-SEM image of ZIF-8 coated with PDA nanospheres in the absence of TMB.

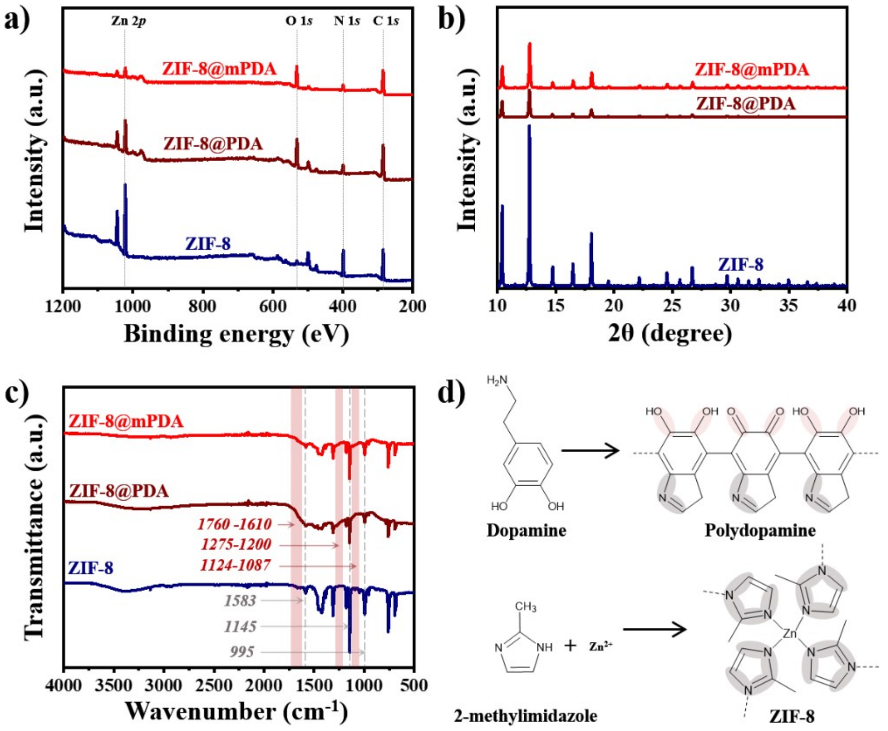

Figure S3. (a) Wide-range X-ray photoelectron spectroscopy (XPS), (b) X-ray diffraction (XRD) patterns and (c) Fourier-transform infrared (FTIR) spectra of ZIF-8, ZIF-8@PDA, ZIF-8@mPDA. (d) Molecular structures of dopamine, polydopamine, 2-methylimidazole, ZIF-8. C-O/C=O bonds are highlighted in pink, while C-N/C=N bonds are highlighted in grey.

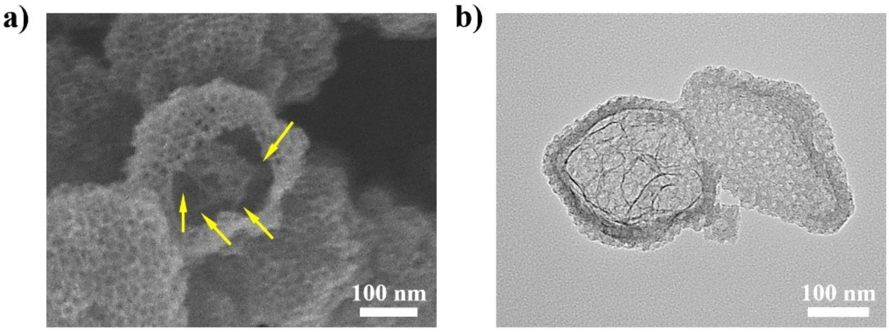

Figure S4. (a) FE-SEM and (b) Transmission electron microscopy (TEM) images of broken HPC. Yellow arrows indicate the carbon scaffolds connecting outer and inner shells.

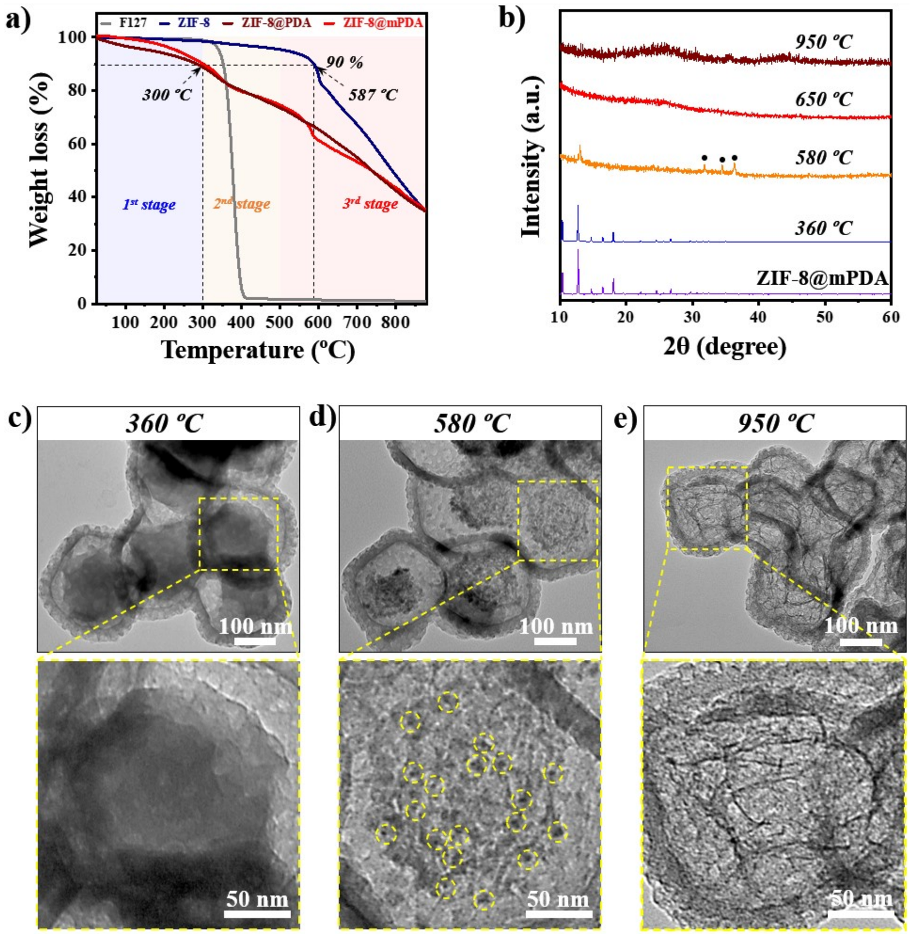

Figure S5. (a) Thermal gravimetry analysis (TGA) graphs of F127, ZIF-8 and ZIF-8@mPDA. (b) XRD spectra of ZIF-8@mPDA carbonized at 360, 580, 650 and 950 °C. TEM images of ZIF-8@mPDA carbonized at (c) 360 °C, (d) 580 °C and (e) 950 °C and the enlarged image of the respective core. The black dots in XRD spectrum (orange) indicate the peak positions of ZnO. The yellow dotted-circles in (d) indicate ZnO particles.

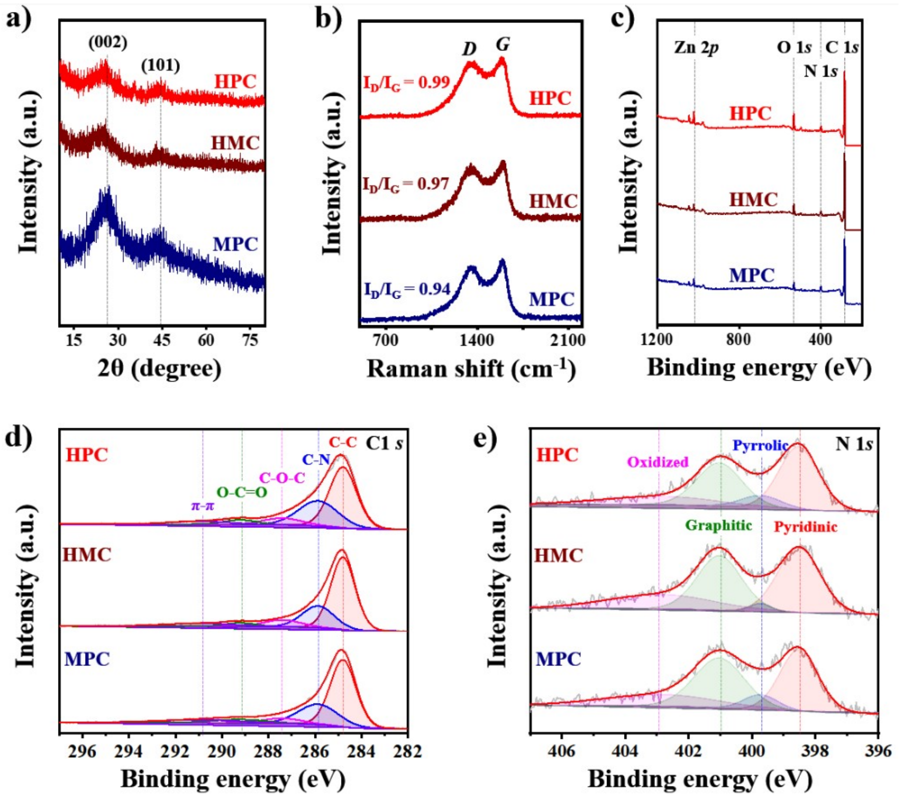

Figure S6. (a) XRD, (b) Raman and (c) XPS spectra of MPC (navy), HMC (brown) and HPC (red). High-resolution XPS spectra of MPC, HMC and HPC for (d) C 1*s* and (e) N 1*s*.

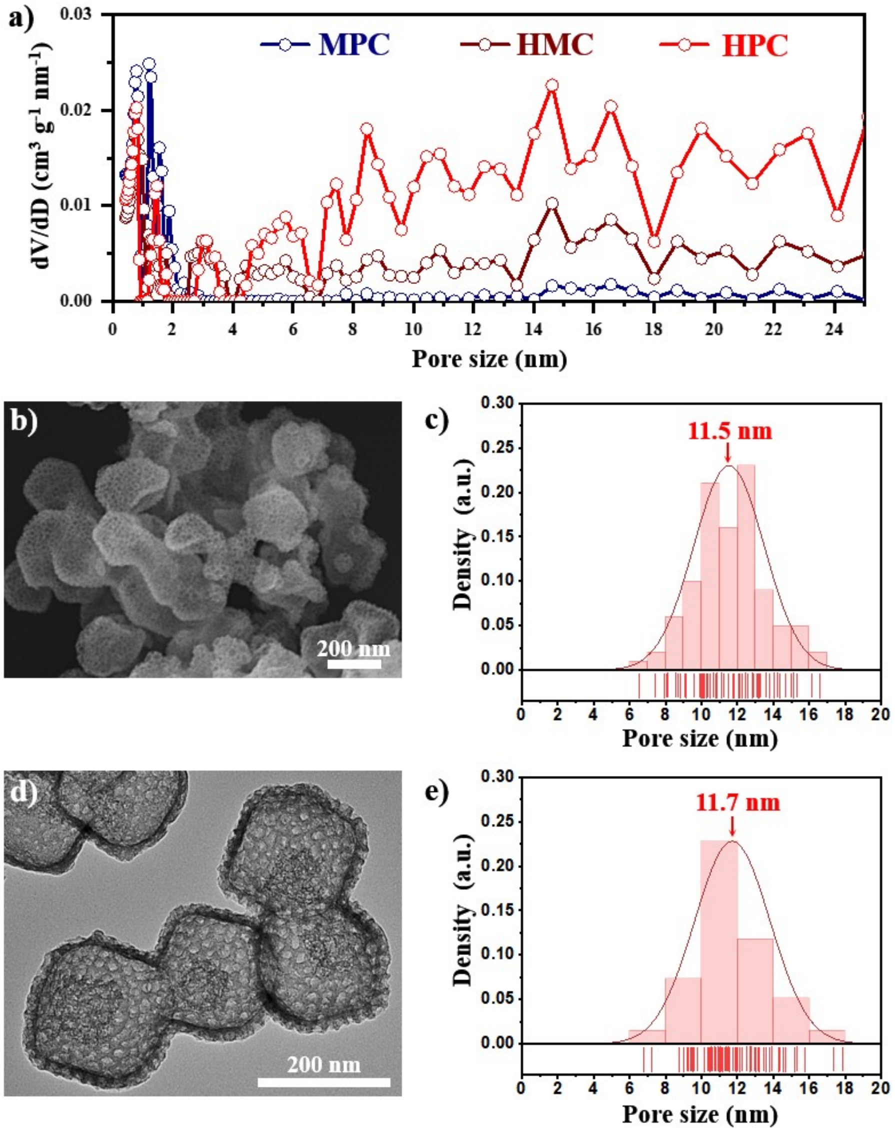

Figure S7. (a) NLDFT pore size distribution of MPC, HMC and HPC. (b) FE-SEM image of HPC and (c) the respective pore size distribution. (d) TEM image of HPC and (e) the respective pore size distribution.

Supplementary note on Figure S7: Due to the very thin shell thickness of HPC, the NLDFT pore size distribution curve (**Fig. S7a**) cannot clearly detect the mean size of mesopores in the mesoporous outer shells.

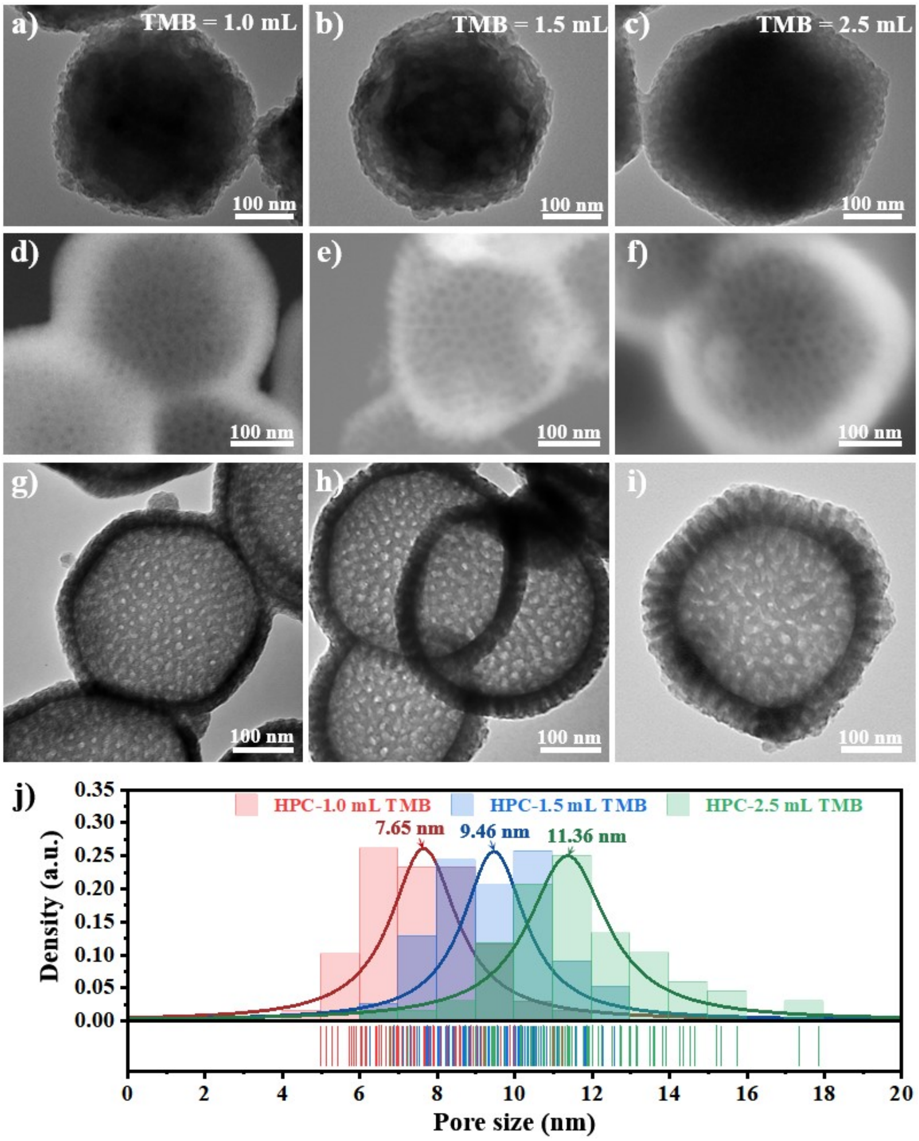

Figure S8. TEM images of (a) ZIF-8@mPDA-1.0 mL TMB, (b) ZIF-8@mPDA-1.5 mL TMB and (c) ZIF-8@mPDA-2.5 mL TMB. SEM images of (d) HPC-1.0 mL TMB, (e) HPC-1.5 mL TMB and (f) HPC-2.5 mL TMB. TEM images of (g) HPC-1.0 mL TMB, (h) HPC-1.5 mL TMB and (i) HPC-2.5 mL TMB. (j) Pore size distributions of HPC-1.0 mL TMB, HPC-1.5 mL TMB and HPC-2.5 mL TMB.

Supplementary note on Figure S8:

To better visualize the mesopores on the outer shell of HPC, ZIF-8 core in ZIF-8@mPDA particle was first removed by 0.1 M H_2SO_4 prior to the direct-carbonization.

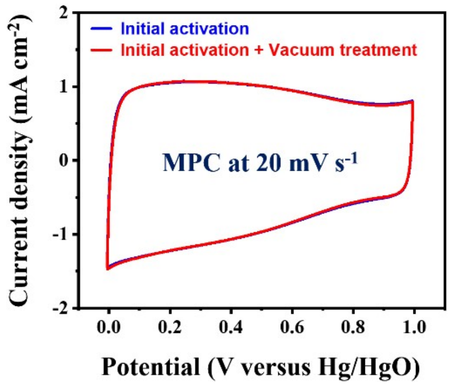

Figure S9. Cyclic voltammetry curves of MPC at 20 mV s^{-1} after initial activation (Blue) and the combination of initial activation and subsequent vacuum treatment (Red).

Supplementary note on Figure S9 and initial electrochemical activation:

Figure S9 represents the CV curves of MPC electrode at 20 mV s^{-1} obtained after the initial activation and the combination of initial activation. As both CV curves overlay on each other with no significant increase or decrease in current density over the potential window, we confirm that the initial activation by 200 consecutive CV cycles at 20 mV s^{-1} is sufficient to electrochemically activate MPC at a full extent. As MPC that has the highest specific surface area and narrowest pore size distribution with majority of its pores lie in micropore range, we conclude that the initial activation by 200 consecutive CV cycles at 20 mV s^{-1} is also sufficient to fully activate other types of porous carbon samples in this study. Therefore, we minimize the effect of the initial contact wettability of the carbon samples to the negligible level for the electrochemical analysis in this study.

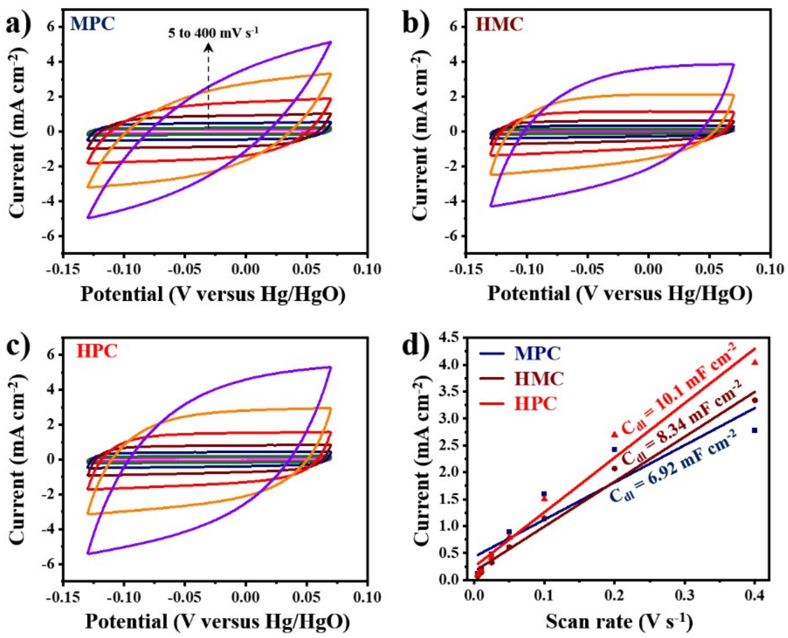

Figure S10. Cyclic voltammetry (CV) curves of (a) MPC, (b) HMC and (c) HPC at a range of scan rates from 5 to 400 mV s^{-1}. (d) Linear fitted lines of anodic currents measured at specific scan rates from 5 to 400 mV s^{-1}.

Supplementary note on Figure S10 and electrochemically active surface area (ECSA):

To calculate ECSA of carbon sample, CV curves were first obtained at a non-Faradaic region (-0.13 to 0.07 V, with open circuit potential roughly at the center of the potential window) with scan rates at 5, 10, 25, 50, 100 and 200 mV s^{-1} (**Figure S7a-c**). The anodic currents at -0.025 V of the CV curves were plotted as a function of scan rates, and the slope of linear fitted line was obtained (**Figure S7d**). The slope indicates the double-

layer capacitance (C_{dl}) of the carbon sample. Typically, C_{dl} values of MPC, HMC and HPC are 11.93, 10.22 and 13.32 mF cm^{-2}, respectively (R-values of linear fitted lines for MPC, HMC and HPC are 0.973, 0.996 and 0.998, respectively). ECSA can then be calculated with the given C_{dl} values using the specific capacitance value of a flat and smooth surface with the geometrical surface area of 1 cm^2. The specific capacitance for the flat and smooth surface is generally found to be from 0.02 to 0.06 mF cm^{-2}, and we assume its value to be 0.04 mF cm^{-2} to calculate ECSA in this study.

ECSA can be calculated using the following equation,

$$ECSA = \frac{C_{dl}}{0.04\, mF\, cm^{-2}}$$

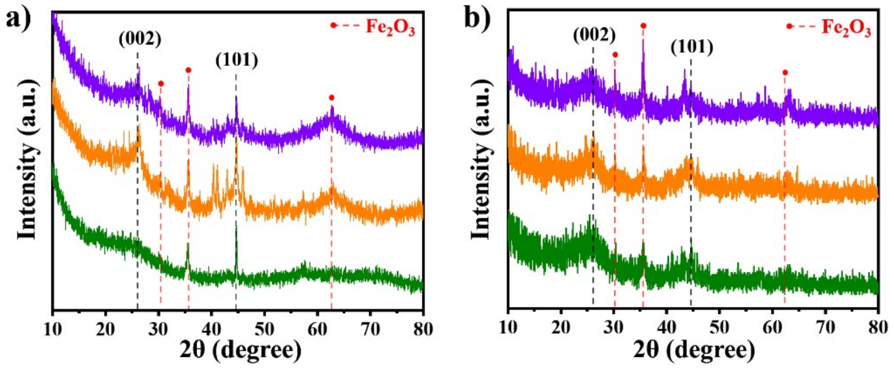

Figure S11. (a) XRD spectra of FeNP- HPC (green), FeNP-HPC-200 (orange) and FeNP-HPC-400 (purple) and (b) that of Fe/N-HPC (green), Fe/N-HPC-200 (orange) and Fe/N-HPC-400 (purple).

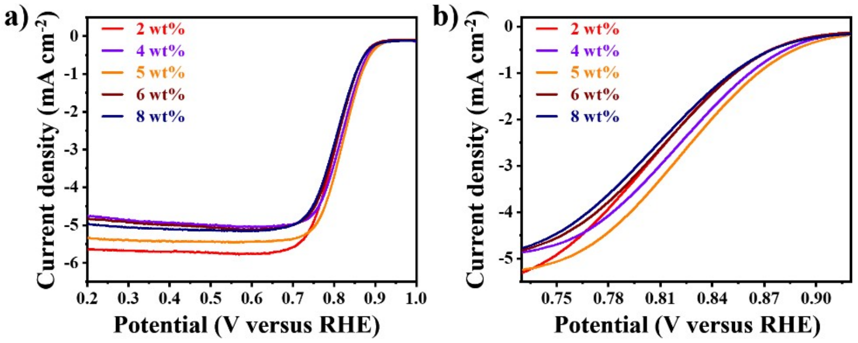

Figure S12. (a) Linear sweep voltammetry (LSV) curves of Fe/N-HPCs prepared with different Fe loading amounts (2, 4, 5, 6 and 8 wt%) and (b) the enlarged LSV curves at the potential window between 0.73 and 0.92 V (*vs* RHE) in O_2 saturated 0.1 M KOH solution.

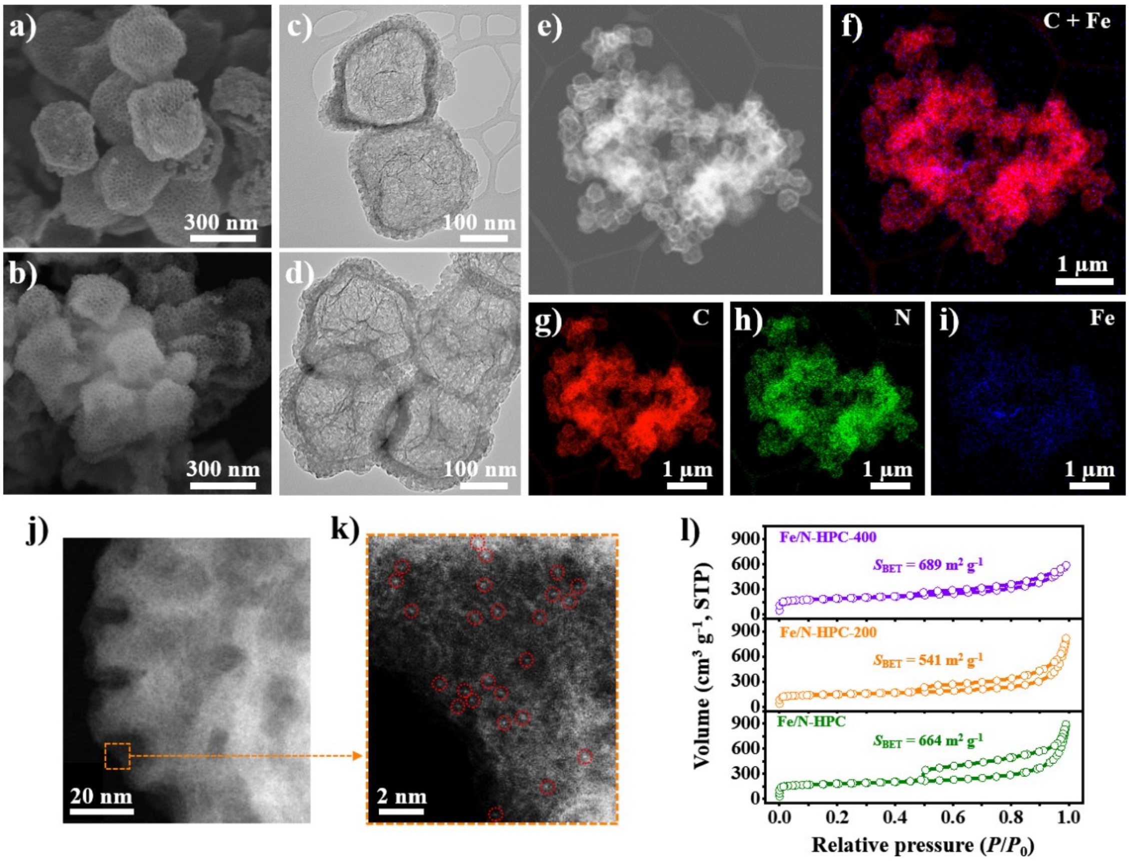

Figure S13. FE-SEM images of (a) Fe/N-HPC and (b) Fe/N-HPC-400. TEM images of (c) Fe/N-HPC and (d) Fe/N-HPC-400. (e) STEM image of Fe/N-HPC-200, STEM-EDS images of (f) overlaid C and Fe atoms, (g) C atoms, (h) N atoms and (i) Fe atoms in Fe/HPC-200. (j, k) HADDF-STEM images of Fe/N-HPC-200. (l) N_2 adsorption/desorption isotherms of Fe/N-HPC, Fe/N-HPC-200 and Fe/N-HPC-400. Red circles in k) indicate individual single Fe atoms.

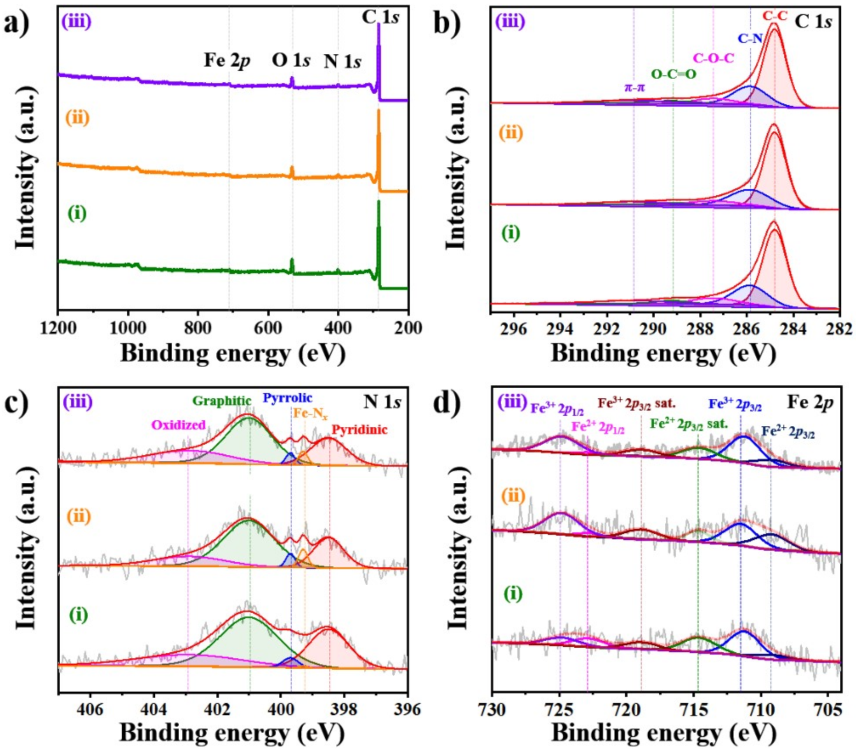

Figure S14. (a) XPS spectra of (i) Fe/N-HPC, (ii) Fe/N-HPC-200 and (iii) Fe/N-HPC-400. High-resolution XPS spectra for (b) C 1*s*, (c) N 1*s* and (d) Fe 2*p* of Fe/N-HPC, Fe/N-HPC-200 and Fe/N-HPC-400.

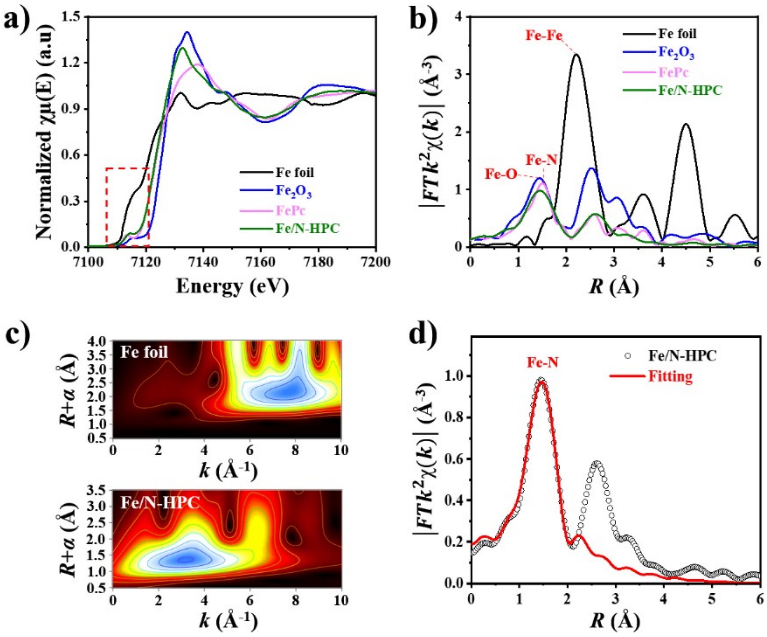

Figure S15. (a) XANES and (b) FT-EXAFS curves of Fe/N-HPC-200 and references at Fe K-edge. (c) WT-EXAFS of Fe foil and Fe/N-HPC. (d) FT-EXAFS fitting curve of Fe/N-HPC.

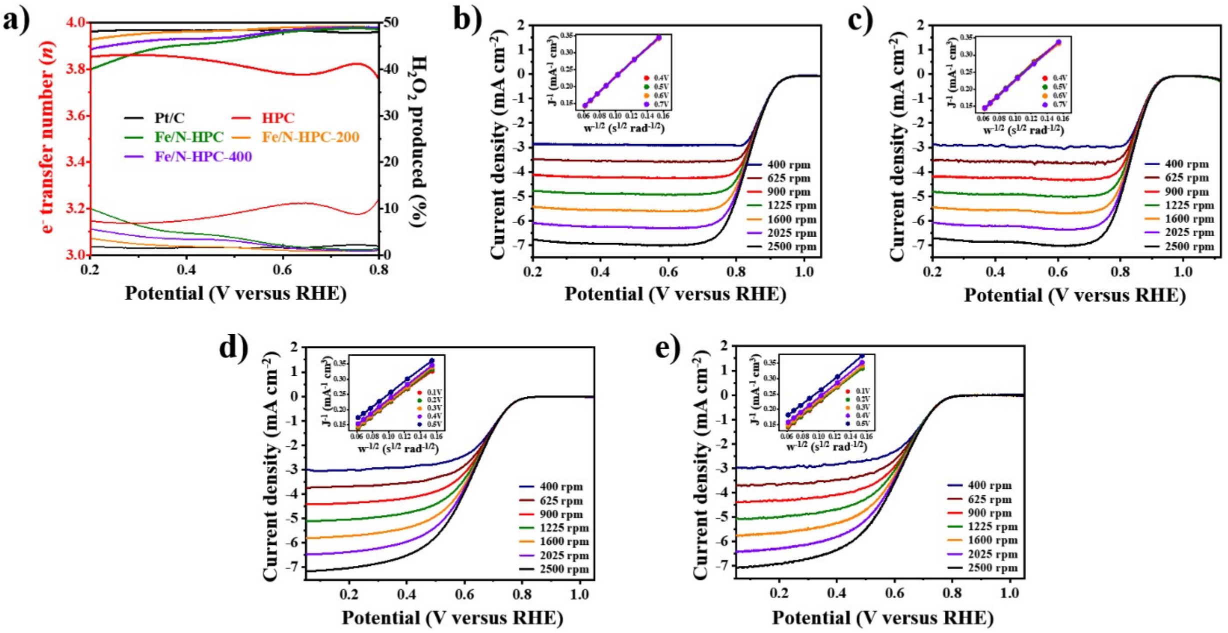

Figure S16. (a) Electron transfer number and H_2O_2 yield of Pt/C, HPC, Fe/N-HPC, Fe/N-HPC-200, and Fe/N-HPC-400. LSV curves at different rotating speeds for (b) Fe/N-HPC-200 and (c) Fe/N-HPC-400 in O_2-saturated 0.1 M KOH electrolyte (Inset: the corresponding K-L plots). LSV curves at different rotating speeds for (d) Fe/N-HPC-200 and (e) Fe/N-HPC-400 in O_2-saturated 0.1 M $HClO_4$ electrolyte. (Inset: the corresponding K-L plots)

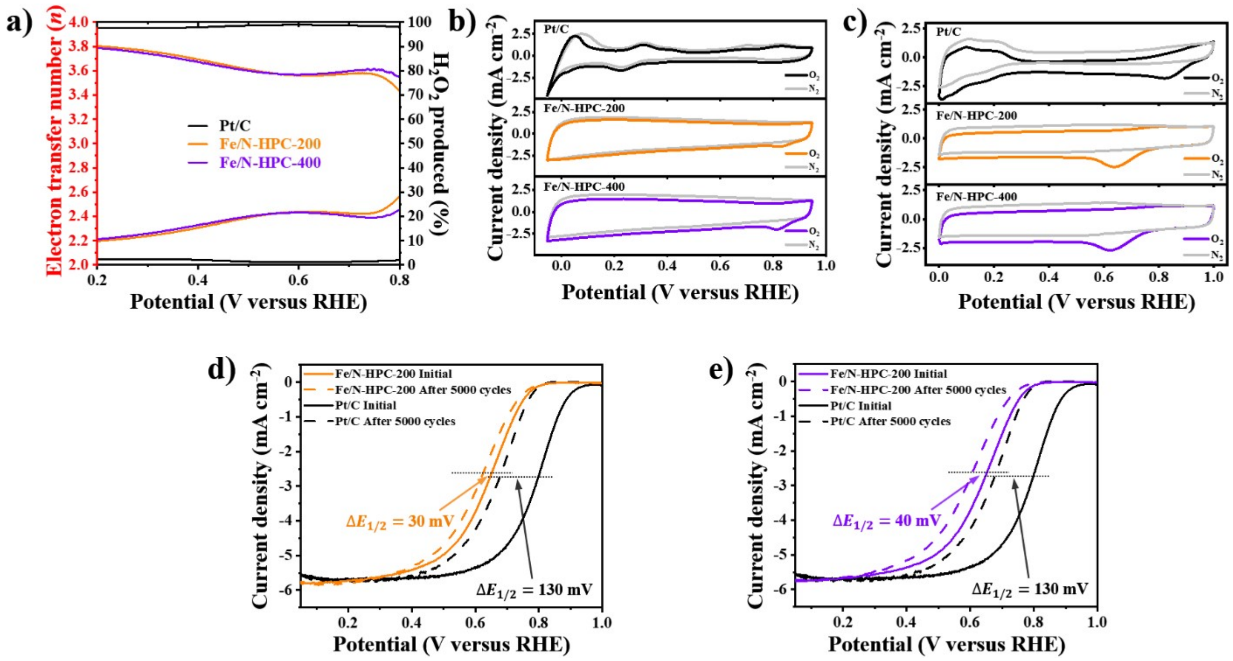

Figure S17. (a) Electron transfer number and H_2O_2 yield of Pt/C, Fe/N-HPC-200, and Fe/N-HPC-400. (b) CV curves of Pt/C, Fe/N-HPC-200, and Fe/N-HPC-400 in O_2-saturated 0.1 M KOH electrolyte and (c) that in O_2-saturated 0.1 M $HClO_4$ electrolyte. (d) LSV curves of Pt/C and Fe/N-HPC-200 before and after the ADT in O_2-saturated 0.1 M $HClO_4$ electrolyte. (e) LSV curves of Pt/C and Fe/N-HPC-400 before and after the ADT in O_2-saturated 0.1 M HClO4 electrolyte. Solid and dotted lines indicate 1st cycle and 5000th cycle, respectively.

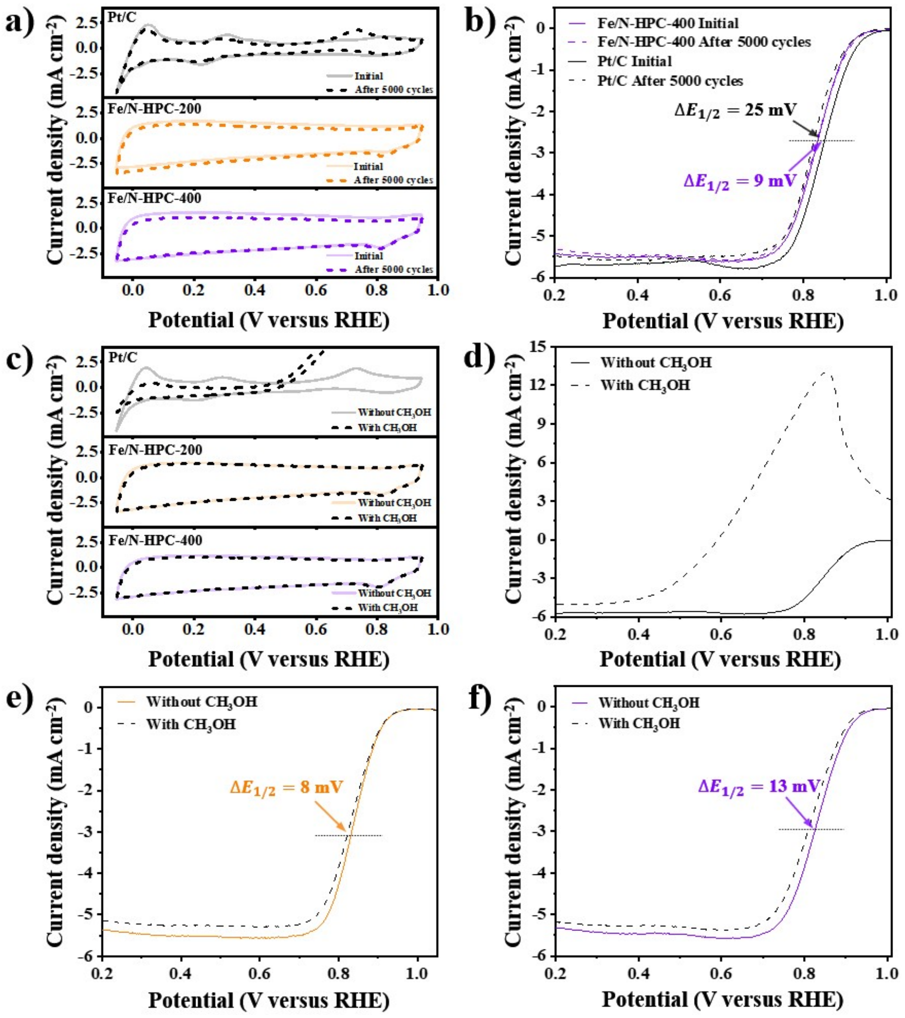

Figure S18. (a) Cyclic voltammetry (CV) curves of Pt/C, Fe/N-HPC-200, and Fe/N-HPC-400 before and after accelerated durability test (ADT). (b) LSV curves of Pt/C and Fe/N-HPC-400 before and after ADT. Solid and dotted lines indicate 1st cycle and 5000th cycle, respectively. (c) CV curves of Pt/C, Fe/N-HPC-200, and Fe/N-HPC-400 before and after the addition of 3 M methanol. LSV curves of (d) Pt/C, (e) Fe/N-HPC-200 and (f) Fe/N-HPC-400 before and after the addition of 3 M methanol. Dotted curves were obtained after the addition of 3 M methanol. All data were obtained in O_2-saturated 0.1 M KOH electrolyte.

Table S1. Elemental content of oxygen, carbon, nitrogen and zinc in all samples from XPS.

	O (at%)	C (at%)	N (at%)	Zn (at%)
ZIF-8	3.20	59.1	26.3	11.4
ZIF-8@PDA	20.0	66.8	8.96	4.24
ZIF-8@mPDA	21.1	69.5	7.41	1.99
ZIF-8@mPDA-200	22.2	68.1	7.97	1.73
ZIF-8@mPDA-400	20.3	67.7	8.94	3.06

Table S2. Elemental content of oxygen, carbon, nitrogen, zinc and iron in all carbon samples from XPS.

	O (at%)	C (at%)	N (at%)	Zn (at%)	Fe (at%)
MPC	4.79	88.0	5.88	1.33	0.00
HMC	5.36	89.4	4.65	0.59	0.00
HPC	7.99	86.0	4.89	1.12	0.00
Fe/N-HPC	6.46	89.98	3.26	0.00	0.30<br>(1.34 wt%)
Fe/N-HPC-200	6.08	89.84	3.79	0.00	0.29<br>(1.30 wt%)
Fe/N-HPC-400	5.75	90.77	3.18	0.00	0.30<br>(1.34 wt%)

Supplementary note on Table S2: wt% of Fe in Fe/N-HPC, Fe/N-HPC-200 and Fe/N-HPC-400 are derived from the at% from XPS by following equation:

$$wt\%\ of\ Fe = \frac{(at\%\ of\ Fe\ \times 55.845)}{(at\%\ of\ O \times 15.999) + (at\%\ of\ C \times 12.011) + (at\%\ of\ N \times 14.007) + (at\%\ of\ Fe \times 55.845)} \times 100\%$$

Table S3. Percentage of C-C, C-N, O-C-O, O-C=O and π-π of C 1*s* derived from high-resolution XPS.

	C-C (at%)	C-N (at%)	O-C-O (at%)	O-C=O (at%)	π-π (at%)
MPC	49.4	19.9	7.82	3.77	7.11
HMC	51.8	20.6	7.55	3.28	6.17
HPC	43.7	26.2	7.09	4.07	4.94
Fe/N-HPC	53.3	19.4	7.62	3.03	6.59
Fe/N-HPC-200	53.7	20.2	6.69	2.67	6.61
Fe/N-HPC-400	54.5	19.2	6.55	4.13	7.14

Table S4. Percentage of pyridinic, pyrrolic, graphitic and oxidized N of N 1*s* derived from high-resolution XPS.

	Pyridinic N (at%)	Pyrrolic N (at%)	Graphitic N (at%)	Oxidized N (at%)
MPC	2.02	0.45	2.18	1.23
HMC	1.84	0.12	1.63	1.06
HPC	2.04	0.41	1.61	0.83

Table S5. Brunauer-Emmett-Teller (BET) specific surface area (S_{BET}), total pore volume (V_{Pore}), micropore volume (V_{Micro}) and mesopore volume (V_{Meso}).

	S_{BET} (m^2 g^{-1})	V_{Pore} (cm^3 g^{-1})	V_{Micro} (cm^3 g^{-1})	V_{Meso} (cm^3 g^{-1})
MPC	889	0.651	0.452	0.090
HMC	696	0.749	0.290	0.260
HPC	721	1.33	0.206	0.330
Fe/N-HPC	664	1.34	0.160	0.298
Fe/N-HPC-200	541	1.25	0.131	0.247
Fe/N-HPC-400	689	0.901	0.219	0.390

Table S6. Electrocatalytic ORR performances of carbon-based catalysts in alkaline electrolyte.

	$E_{1/2}$ (V vs RHE)	E_{onset} (V vs RHE)	*n* (*RRDE*)	H_2O_2 yield (%)
MPC	0.71	0.82	3.54	23.0
HMC	0.70	0.82	3.68	16.1
HPC	0.72	0.82	3.82	8.80
Fe/N-HPC	0.82	0.92	3.92	3.94
Fe/N-HPC-200	0.85	0.96	3.97 (*K-L*: 3.96)	1.90
Fe/N-HPC-400	0.84	0.95	3.95 (*K-L*: 3.99)	3.14
Pt/C	0.85	0.97	3.97	1.70

Table S7. Percentage of pyridinic, Fe-N_x, pyrrolic, graphitic and oxidized N of N 1*s* derived from high-resolution XPS.

	Pyridinic N (at%)	Fe-N_x (at%)	Pyrrolic N (at%)	Graphitic N (at%)	Oxidized N (at%)
Fe/N-HPC	0.88	0.01	0.09	1.61	0.67
Fe/N-HPC-200	0.82	0.15	0.11	2.11	0.60
Fe/N-HPC-400	0.57	0.07	0.06	1.22	0.55

Table S8. EXAFS fitting results of Fe foil, Fe/N-HPC and Fe/N-HPC-200

	Shell	N	R	σ^2	R factor (%)
Fe foil[a]	Fe-Fe	8	2.48	0.008	1.68
	Fe-Fe	6	2.83	0.008	1.68
Fe/N-HPC[b]	Fe-N	4.4±0.4	2.00	0.010	0.13
Fe/N-HPC-200[c]	Fe-N	2.8±0.7	2.01	0.003	1.59

N, coordination number; R, distance between absorber and backscatter atoms; σ^2, Debye-Waller factor accounting for thermal and structural disorders; R factor (%), the goodness of the fit. [a] Fitting range: Fe foil: 3.09≤k (/Å)≤12.15 and 1.34≤R (Å)≤3.00. [b] Fitting range: Fe/N-HPC:2.71≤k (/Å)≤8.98 and 1.10≤R (Å)≤2.10. [c] Fitting range: Fe/N-HPC-200:2.80≤k (/Å)≤6.70 and 1.10≤R (Å)≤2.11

Table S9. Comparison table for the ORR catalytic activity of Fe/N-HPC-200 with the previously reported ORR catalysts in O_2-saturated 0.1 M KOH electrolyte.

	Loading amount of the catalyst (mg cm^{-2})	$E_{1/2}$ (V vs RHE)	E_{onset} (V vs RHE)	*n* (RRDE)	Reference
Fe/N-HPC-200	0.2	0.85	0.96	3.97 (K-L: 3.96)	This work
Fe-ISAs/H-CN	0.637	0.87	n.a.	(K-L: 3.9)	5
Fe-NCNT	0.35	0.860	0.987	(K-L: 3.92)	6
Fe/N-GPC	0.4	0.86	0.98	(K-L: ~4)	7
Fe/N/C	0.8	0.87	0.98	~4	8
Fe-N/C	0.8	0.865	n.a.	n.a.	9
Fe doped MOF CoV@CoO	0.5	n.a.	0.86	n.a.	10
CoN/FeN@N,S-C-800	0.48	0.865	0.998	3.88 (K-L: ~4)	11
FeN_2/NOMC	0.5	0.863	1.05	(K-L: ~4)	12
Fe_3C-GNR	0.14	0.78	0.8	(K-L: ~3.9)	13
C-Fe-Z8-15	0.35	0.843	0.926	(K-L: ~3.6)	14
Fe-doped MOF CuCoSe	n.a.	0.756	n.a.	(K-L: ~4)	15
PMF-800	1.2	0.861	n.a.	3.99	16
Fe_3C/NG-800	0.4	0.86	1.03	3.89-4.0	17

Reference

1 S. H. Lee, J. Kim, D. Y. Chung, J. M. Yoo, H. S. Lee, M. J. Kim, B. S. Mun, S. G. Kwon, Y.-E. Sung, T. Hyeon, *J. Am. Chem. Soc.* 2019, ***141*** (5), 2035-2045.

2 J. H. Jang, S. M. Oh, *J. Electrochem. Soc.* 2004, ***151*** (4), A571.

3 D. D. Macdonald, M. Urquidi-Macdonald, *J. Electrochem. Soc.* 1985, ***132*** (10), 2316-2319.

4 M. Urquidi-Macdonald, S. Real, D. D. Macdonald, *J. Electrochem. Soc.* 1986, ***133*** (10), 2018-2024.

5 C. Wang, Y. Chen, M, Zhong, T. Feng, Y. Liu, S. Feng, N. Zhang, L. Shen, K. Zhang, B. Yang, *J. Mater. Chem. A* 2021, ***9***, 22095-22101.

6 Y. Guo, A. Dong, Q. Huang, Q. Li, Y. Hu, J. Qian, S. Huang, *J. Colloid Interface Sci.*, 2022, ***606***, 1833-1841.

7 Q.-L. Zhu, W. Xia, L.-R. Zheng, R. Zou, Z. Liu, Q. Xu, *ACS Energy Lett.* 2017, ***2***, 504-511.

8 Q. Wei, G. Zhang, X. Yang, Y. Fu, Q. Yang, N. Chen, W. Chen, S. Sun, *J. Mater. Chem. A* 2018, ***6***, 4605-4610.

9 S. Gong, C. Wang, P. Jiang, L. Hu, H. Lei, Q. Chen, *J. Mater. Chem. A* 2018, ***6***, 13254-13262.

10 A. Muthurasu, A. P. Tiwari, K. Chhetri, B. Dahal, H. Y. Kim, *Nano Energy* 2021, ***88***, 106238.

11 S. Ren, X. Duan, M. Lei, S. Liang, M. Zhang, H. Zheng, *Appl. Surf. Sci.* 2021, ***569***, 151030.

12 H. Shen, E. Gracia-Espino, J. Ma, H. Tang, X. Mamat, T. Wagberg, G. Hu, S. Guo, *Nano Energy* 2017, ***35***, 9-16.

13 X. Fan, Z. Peng, R. Ye, H. Zhou, X. Guo, *ACS Nano* 2015, ***9***, 7407-7418.

14 X. Zhao, M. Chen, Z. Zuo, J. Wang, Z. Liu, L. Wang, B. Liu, G. Wu, H. Zhang, H. Yang, *J. Electrochem. Soc.* 2018, ***165***, F1279.

15 S.-H. Chae, A. Muthurasu, T. Kim, J. S. Kim, M.-S. khil, M. Lee, H. Kim, J. Y. Lee, H. Y. Kim, *Appl. Catal., B* 2021, ***293***, 120209.

16 W. Yang, X. Liu, X. Yue, J. Jia, S. Guo, *J. Am. Chem. Soc.* 2015, ***137***, 1436-1439.

17 M. Xiao, J. Zhu, L. Feng, C. Liu, W. Xing, *Adv. Mater.* 2015, ***27***, 2521-2527.
